# Using Passive Sensing to Predict Psychosis Relapse: An In-Depth Qualitative Study Exploring Perspectives of People With Psychosis

**DOI:** 10.1093/schbul/sbaf126

**Published:** 2025-08-21

**Authors:** Emily Eisner, Hannah Ball, John Ainsworth, Matteo Cella, Natalie Chalmers, Sybil Clifford, Richard J Drake, Daniel Elton, Sophie Faulkner, Kathryn Greenwood, Andrew Gumley, Gillian Haddock, Kimberley M Kendall, Alex Kenny, Tor-Ivar Krogsæter, Jane Lees, Shôn Lewis, Laura Maclean, Kathryn O’Hare, Alie Phiri, Cara Richardson, Matthias Schwannauer, Rebecca Turner, Annabel Walsh, James Walters, Til Wykes, Uzma Zahid, Sandra Bucci

**Affiliations:** Division of Psychology and Mental Health, Faculty of Biology, Medicine and Health, Manchester Academic Health Science Centre, School of Health Sciences, The University of Manchester, Manchester, Lancashire, M13 9PL, United Kingdom; Greater Manchester Mental Health NHS Foundation Trust, Prestwich, Manchester, M25 3BL, United Kingdom; Division of Psychology and Mental Health, Faculty of Biology, Medicine and Health, Manchester Academic Health Science Centre, School of Health Sciences, The University of Manchester, Manchester, Lancashire, M13 9PL, United Kingdom; Greater Manchester Mental Health NHS Foundation Trust, Prestwich, Manchester, M25 3BL, United Kingdom; Division of Informatics, Imaging and Data Sciences, Faculty of Biology, Medicine and Health, Manchester Academic Health Science Centre, School of Health Sciences, University of Manchester, Manchester, United Kingdom; Department of Psychology, Institute of Psychiatry, Psychology & Neuroscience, King’s College London, London, United Kingdom; South London & Maudsley NHS Foundation Trust, London Hospital, Denmark Hill, London, SE5 8AZ, United Kingdom; School of Health in Social Science, University of Edinburgh, Edinburgh, EH8 9AG, United Kingdom; School of Psychology, University of Sussex, Falmer, Brighton BN1 9QH, United Kingdom; Division of Psychology and Mental Health, Faculty of Biology, Medicine and Health, Manchester Academic Health Science Centre, School of Health Sciences, The University of Manchester, Manchester, Lancashire, M13 9PL, United Kingdom; Greater Manchester Mental Health NHS Foundation Trust, Prestwich, Manchester, M25 3BL, United Kingdom; The McPin Foundation, London, E2 9DA, United Kingdom; Division of Psychology and Mental Health, Faculty of Biology, Medicine and Health, Manchester Academic Health Science Centre, School of Health Sciences, The University of Manchester, Manchester, Lancashire, M13 9PL, United Kingdom; Greater Manchester Mental Health NHS Foundation Trust, Prestwich, Manchester, M25 3BL, United Kingdom; School of Psychology, University of Sussex, Falmer, Brighton BN1 9QH, United Kingdom; Research and Development Department, Sussex Partnership NHS Foundation Trust, Hove, BN3 7HZ, United Kingdom; School of Health and Wellbeing, University of Glasgow, Glasgow, G12 8TB, United Kingdom; NHS Greater Glasgow & Clyde, Glasgow, G12 0XH, United Kingdom; Division of Psychology and Mental Health, Faculty of Biology, Medicine and Health, Manchester Academic Health Science Centre, School of Health Sciences, The University of Manchester, Manchester, Lancashire, M13 9PL, United Kingdom; Greater Manchester Mental Health NHS Foundation Trust, Prestwich, Manchester, M25 3BL, United Kingdom; MRC Centre for Neuropsychiatric Genetics and Genomics, Division of Psychological Medicine and Clinical Neurosciences, Cardiff University, Cardiff, CF24 4HQ, United Kingdom; The McPin Foundation, London, E2 9DA, United Kingdom; The McPin Foundation, London, E2 9DA, United Kingdom; Division of Psychology and Mental Health, Faculty of Biology, Medicine and Health, Manchester Academic Health Science Centre, School of Health Sciences, The University of Manchester, Manchester, Lancashire, M13 9PL, United Kingdom; Division of Psychology and Mental Health, Faculty of Biology, Medicine and Health, Manchester Academic Health Science Centre, School of Health Sciences, The University of Manchester, Manchester, Lancashire, M13 9PL, United Kingdom; Greater Manchester Mental Health NHS Foundation Trust, Prestwich, Manchester, M25 3BL, United Kingdom; School of Health in Social Science, University of Edinburgh, Edinburgh, EH8 9AG, United Kingdom; School of Health and Wellbeing, University of Glasgow, Glasgow, G12 8TB, United Kingdom; The McPin Foundation, London, E2 9DA, United Kingdom; Division of Psychology and Mental Health, Faculty of Biology, Medicine and Health, Manchester Academic Health Science Centre, School of Health Sciences, The University of Manchester, Manchester, Lancashire, M13 9PL, United Kingdom; School of Health in Social Science, University of Edinburgh, Edinburgh, EH8 9AG, United Kingdom; NHS Lothian, Edinburgh, EH3 9DN, United Kingdom; Division of Psychology and Mental Health, Faculty of Biology, Medicine and Health, Manchester Academic Health Science Centre, School of Health Sciences, The University of Manchester, Manchester, Lancashire, M13 9PL, United Kingdom; The McPin Foundation, London, E2 9DA, United Kingdom; MRC Centre for Neuropsychiatric Genetics and Genomics, Division of Psychological Medicine and Clinical Neurosciences, Cardiff University, Cardiff, CF24 4HQ, United Kingdom; Department of Psychology, Institute of Psychiatry, Psychology & Neuroscience, King’s College London, London, United Kingdom; South London & Maudsley NHS Foundation Trust, London Hospital, Denmark Hill, London, SE5 8AZ, United Kingdom; Department of Psychology, Institute of Psychiatry, Psychology & Neuroscience, King’s College London, London, United Kingdom; Division of Psychology and Mental Health, Faculty of Biology, Medicine and Health, Manchester Academic Health Science Centre, School of Health Sciences, The University of Manchester, Manchester, Lancashire, M13 9PL, United Kingdom; Greater Manchester Mental Health NHS Foundation Trust, Prestwich, Manchester, M25 3BL, United Kingdom

**Keywords:** digital phenotyping, active symptom monitoring, personal sensing, machine learning, qualitative

## Abstract

**Background:**

Relapses result in negative consequences for individuals with psychosis and considerable health service costs. Digital remote monitoring (DRM) systems incorporating “passive sensing” (sensor data gathered via smartphones/wearables) may be a low-burden method for identifying relapses early, enabling prompt intervention and potentially averting the consequences of full relapse.

**Objective:**

This study examined detailed views from people with psychosis about using passive sensing in this context.

**Study Design:**

Qualitative interviews, analyzed using reflexive thematic analysis. Setting: Secondary care mental health services across the United Kingdom. An advisory group with relevant lived experience was involved throughout, from developing the topic guide to analysis. Participants: Clinician confirmed diagnosis of schizophrenia-spectrum psychosis (*n* = 58).

**Study Results:**

Four overarching themes were developed. Theme 1 outlined participants’ polarized feelings about passive sensing, highlighting specific challenges relating to privacy, especially regarding location data. Theme 2 examined participants’ fears that clinicians might judge their movements or routines, creating a sense of pressure to modify their actions and undermining their autonomy. Theme 3 described potential solutions: offering users choice about what data are shared, when, and with whom. Theme 4 outlined specific benefits that participants valued, including intended functions of passive sensing within DRM (ease of use, early identification of relapse, and relevance of sleep monitoring) and novel uses.

**Conclusions:**

Our findings underline the importance of fully informed consent, choice, and autonomy. Given the potential privacy impacts, individuals are unlikely to engage with passive sensing unless they perceive clear personal benefits. Prospective DRM users need clear, accessible information about passive data collection and its relevant costs and benefits.

## Introduction

Although around half of people experiencing a first episode of psychosis achieve symptom remission,^1^ half relapse within 3 years.^2^ Relapses are associated with considerable setbacks for individuals (eg, disrupted education/employment),^3^^,^^4^ worse functioning,^4^^,^^5^ poorerclinical outcomes,^6^^,^^7^ greater mortality risk,^8–10^ and substantial costs for health services, particularly due to hospital admission.^11–14^ Conventional methods of monitoring mental health rely on individuals recalling symptoms from the preceding week(s) or month(s) during infrequent appointments. These retrospective symptom reports lack precision, delaying recognition of emerging relapses. Psychosis requires precise, time-sensitive treatment; thus, the challenge is to identify relapse promptly and offer timely support, without putting further pressure on strained mental health services or people with psychosis.

To address this challenge, our multi-center CONNECT study will develop and test a digital remote monitoring (DRM) system. This DRM system will integrate multimodal data, including “active symptom monitoring” (ASM) via smartphone questionnaires and “passive sensing” using sensor data from smartphones and wearable devices. It will apply a machine learning-generated algorithm to these data to predict psychosis relapse. The CONNECT study output will be a dynamic DRM system that tracks mental health and alerts individuals and/or their clinical team to early signs of relapse, enabling prompt intervention and potentially averting the difficult consequences of a full relapse.

The first study testing an ASM smartphone app for psychotic symptoms was published in 2012^15^; since then, over a decade of research has indicated that ASM is likely to be feasible, acceptable, safe, and valid for people with psychosis.^15–17^ This includes many qualitative investigations examining stakeholders’ views about factors affecting engagement with ASM and implementation in services.^17–19^ Conversely, empirical research testing passive sensing in this group is less established.^20^ Passive sensing uses ongoing smartphone and/or wearable sensor data (eg, Global Positioning System, accelerometer) to estimate social and/or behavioral functioning (eg, inactivity, sleep disturbance) without direct user input.^21^^,^^22^ Hence, a key advantage of passive sensing compared to ASM or more traditional self-report methods is the low user burden. There is some evidence that changes in passive data may be useful for identifying people at risk of imminent relapse. For example, a recent review^21^ identified 8 studies^23–30^ examining behavioral anomalies in the period preceding relapse. However, findings across studies were mixed, and replication in larger samples is needed.^20^^,^^21^ Moreover, in-depth qualitative studies exploring the acceptability of passive sensing to people with psychosis are lacking. Two previous studies briefly discussed this in the context of individuals’ views on DRM systems as a whole, reporting that most participants thought that combining active and passive data was ‘valuable’ and few expressing privacy concerns.^31^^,^^32^ A further two studies have reported brief exit interview data from participants who opted to use an app with a passive monitoring component^33^^,^^34^ these studies had small (*n* = 10-15) and highly selected samples, and the primary focus of the analysis was ASM rather than passive sensing. Hence, no studies to date have explored participants’ in-depth views on passive sensing in a broad sample of people with psychosis. Three studies have examined clinician views, advocating equitable implementation, maintaining human interaction, and ensuring ethical data use to enhance adoption in mental health services.^35–37^

Given the well-documented challenges of implementing new technologies in health services, digital systems should be designed with implementation in mind.^38^^,^^39^ The intended end-users are important stakeholders whose actions determine whether digital systems are successfully adopted^40^; so seeking their views on DRM is essential to ensuring such systems are acceptable and target unmet needs. Hence, we sought detailed views from a large group of people with psychosis, recruited from a range of services across 6 geographically distinct areas of the United Kingdom (UK). Using qualitative interviews, we investigated participants’ hypothetical views about a DRM system that gathers ASM and passive sensing data and applies a machine learning-generated algorithm to predict relapse. Interviews generated a substantial volume of data; therefore, findings related to each DRM component (ASM, passive sensing, and relapse prediction algorithm) will be presented across 3 distinct publications. This ensures a comprehensive, rigorous analysis, allowing in-depth exploration of participants’ nuanced insights and experiences within each component. This article examines detailed participant perspectives on using *passive sensing* within a DRM system for psychosis, exploring key considerations for future integration into existing care pathways across diverse geographical and service settings. This is the first study to date to examine this topic in such detail in a large, nationally distributed sample of participants with psychosis.

## Method

### Design

This qualitative interview study was nested within the multi-center CONNECT study, a Wellcome Trust-funded research program (www.connectdigitalstudy.com). Positioned within Phase 1 of the project, this study informed the development and optimization of the CONNECT DRM system. Relevant approvals were obtained from the Health Research Authority and Research Ethics Committee (REC reference: ^22^/WS/0083). This report follows Consolidated Criteria for Reporting Qualitative Studies guidelines^41^ (Supplementary Material S1).

### Lived Experience Involvement

The CONNECT study has an extensive program of lived experience involvement led by The McPin Foundation. This includes the CONNECT Lived Experience Advisory Panel (LEAP), a diverse group of 12 people with relevant lived experience, recruited from across the 6 study sites. Relevant experience includes past/current experience of psychosis and/or being a family member, friend, carer, or trusted other of someone with past/current experience of psychosis. The CONNECT LEAP advised on ongoing study management and provided feedback on a presentation summarizing the findings. Six people with lived experience of psychosis contributed to the study design and analysis in more depth. Two advised on topic guide wording/flow, including lay descriptions of relevant concepts and interview questions/prompts. Four were directly involved in data analysis and are co-authors of the paper. Having received appropriate training in qualitative research, they coded interview transcripts, advised on the coding structure, sense-checked emerging themes, and reviewed manuscript drafts.

### Participants and Sampling Strategy

Individuals with psychosis were recruited via mental health services in 6 geographical areas of the UK (Manchester, Glasgow, Edinburgh, London, Sussex, and Wales) between November 15, 2022 and November 13, 2023. Eligible individuals were: (1) English speaking; (2) ≥ 16 years old; (3) in current contact with mental health services; (4) capable of informed consent; (5) sufficiently clinically stable to be interviewed; and (6) either had a clinical diagnosis of schizophrenia spectrum disorder (ICD10 F20-29) or met Early Intervention Service entry criteria (operationally defined using the Positive and Negative Syndrome Scale^42^ and/or Comprehensive Assessment of At-Risk Mental States psychosis transition criteria^43^). Participants were purposively sampled from varied geographical locations, ethnicities, ages, mental health service types, clinical presentations, and prior experiences of using digital health tools, smartphones, or wearables. This involved targeted invitations, engagement with different NHS teams, and recruitment across diverse clinical settings to ensure broad representation. Sample size was determined by our aim to recruit approximately 10 participants per site to enable geographical heterogeneity. Informed consent was obtained using a signed paper/electronic consent form or an audio-recorded verbal agreement. To protect anonymity, participants were assigned a unique study identification number. Participants completed demographic questionnaires and received £20 reimbursement.

### Qualitative Interviews

Following informed consent, participants took part in one-off, audio-recorded, individual qualitative interviews, conducted either in-person or via phone call or encrypted videoconferencing software (based on preference and risk assessment). Full interviews lasted 20 to 78 (median 43) minutes and followed a detailed topic guide (Supplementary Material S2). As interviews covered an unfamiliar topic for some, the topic guide included lay descriptions of relevant concepts (eg, passive sensing) and presented figures showing smartphones and wearables, indicating the types of data gathered by each (eg, location, heart rate, and number of calls/texts). Questions on passive sensing explored views about the topic in general and specific data types, including different levels of precision of location data. Descriptions and questions in the topic guide were worded to describe use in a clinical context (rather than a research context). Researchers completed post-interview logs, including brief contextual notes, reflections, and suggested topic guide updates (used to iterate the topic guide). Interviews were transcribed verbatim, anonymized to protect participant confidentiality, and securely stored.

### Data Analysis

Analysis of interview transcripts was inductive, guided by a reflexive thematic analysis approach^44^ comprising: (1) data familiarization, (2) code generation, (3) initial theme creation, (4) revision of themes, (5) defining final themes, and (6) report writing. Alongside inductive coding (stage 2), a small number of research questions were coded deductively (eg, “How frequently should passive sensing consent be renewed?”), following methods used in previous digital health research.^45^ This allowed targeted exploration of predefined implementation issues critical to the ethical and practical integration of DRM systems. To examine whether certain themes related to the passive sensing component of the proposed DRM system, transcript sections were labeled if participants were discussing a specific component or the overall DRM system. Themes related to passive sensing specifically are presented in this paper. Analysis was managed using NVivo qualitative data analysis software.^46^ Researcher E.E. coded all data and integrated 4 LEAP members’ coding of a subset of transcripts. Themes were discussed and refined with input from 4 LEAP members and the wider research team to ensure they were reflective of the original data, related to the aim of the research, grounded in real-world considerations, and told a core interpretative story. Our critical realist epistemological approach^47^^,^^48^ and reflexivity considerations are detailed in Supplementary Material S3.

## Results

### Participant Characteristics

A total of 58 participants were interviewed (see Table 1). Their average age was 39.1 years (SD 14.1). Slightly over half the sample were men, a third were from an ethnic minority group, and three-quarters were single. Roughly half were engaged in employment, voluntary work, or were students, and most had received education post-16 years of age. Primary self-reported diagnosis was typically psychosis or schizophrenia, with current care usually community based. Around half the sample reported having a health or wellbeing app installed on their digital device (details: Supplementary Material S4).

**Table 1:** Demographic Characteristics of the Sample

	**Frequency (*n* = 57)** ^**a**^	**Percentage**
Gender^b^		
Man/male	32	56.1
Woman/female	25	43.9
Ethnicity		
Asian/Asian British	6	10.5
Black African	5	8.8
Black British	4	7.0
Mixed ethnic background	2	3.5
White British	37	64.9
Other ethnic background	3	5.3
Relationship status		
Co-habiting	7	12.3
Divorced or separated	2	3.5
Married	2	3.5
Partnered	4	7.0
Single	42	73.7
Employment status		
Employed	12	21.1
Unemployed (looking for work)	3	5.3
Unemployed (not looking for work)	5	8.8
Retired	3	5.3
Self-employed	6	10.5
Student	3	5.3
Unable to work	19	33.3
Voluntary work	6	10.5
Parental or caring responsibilities	10	17.5
Child(ren)	7	12.3
Family member	1	1.8
Parent(s)	1	1.8
Would rather not say	1	1.8
Education		
Secondary school (GCSEs)	14	24.6
Further education (6th form, college or equivalent vocational education)	21	36.8
University Bachelor’s degree	14	24.6
University Master’s degree	4	7.0
PhD or higher	2	3.5
Other education	1	3.5
Living situation		
Alone	24	42.1
With parents/carers	12	21.1
With partner	8	14.0
With children	3	5.3
With other relatives	1	1.8
With friends or similar	6	10.5
Supported accommodation or similar	3	5.3
Self-reported diagnosis		
Psychosis	22	38.6
Schizophrenia	26	45.6
Schizoaffective	4	7.0
Bipolar	3	5.3
Other	1	1.8
Missing	1	1.8
Current mental health service		
Community mental health team	27	47.4
Early intervention service	15	26.3
Inpatient	5	8.8
Psychiatry outpatients	4	7.0
Forensic outreach team	1	1.8
Rehab or recovery team	2	3.5
None	2	3.5
Missing	1	1.8
App installed on a device^c^	27	47.4
Mental health	13	22.8
Physical health	17	29.8
Wellbeing	9	15.8
Mood and/or health tracking	7	12.3
Other health-related app	3	5.3
None	30	52.6

a Sample size is 58 but all demographics missing for 1 participant (M009).

b All participants reported that their gender matched the sex they were assigned at birth.

c Participants were asked to report which types of app they currently have installed on their digital device (phone, tablet, or wearable). Frequency of app subtypes adds to more than 57 because some people reported more than 1 type of app. See Supplementary Material S4 for details of app names.

### Themes and Subthemes

**Figure 1:**
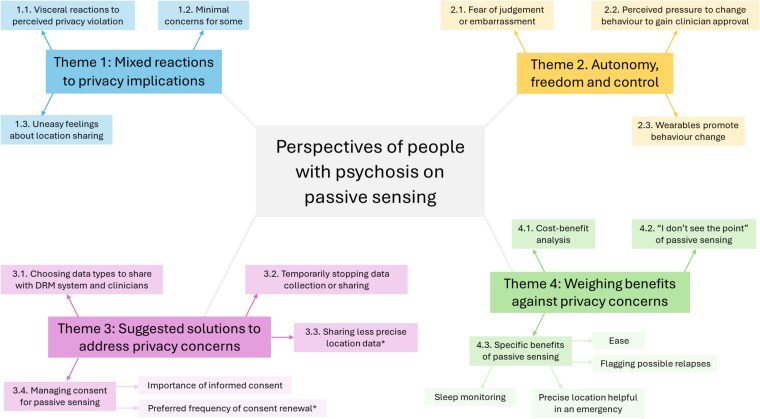
Thematic Map: * indicates deductive subtheme related to an a priori question.

Four themes were developed and are shown on a thematic map (Figure 1). Selected illustrative quotes are shown in Table 2, identified in the main text using letter IDs (eg, “quote X”). Further quotes and a summary of views by data type are shown in Supplementary Material S5 and Material S6. Theme 1 outlines participants’ polarized feelings about passive sensing: some expressed strong negative reactions; others had minimal concerns. Given this contrast, the distribution of strong views on passive sensing was checked against demographic characteristics and geographical location, revealing no clear pattern. Theme 2 examines participants’ beliefs and experiences underlying their privacy concerns and Theme 3 outlines their suggestions for mitigating key concerns. Theme 4 examines the trade-off where participants weighed privacy concerns about passive sensing against its potential usefulness.

**Table 2:** Illustrative Quotes

**Theme and subtheme**	**Quotes**	**ID**
**Mixed reactions to privacy implications**		
Uneasy feelings about location tracking	“In my early years, I’d be like oh my god they’re spying on me, they know my location, they know my text message … With psychosis you go wooop off the wall … Paranoia.” (C007)	A
	“I don’t just feel in control of my situation and life anymore, if an app is to know [my location]. It could give room for mental health problems.” (M002)	B
	“I don’t fancy being tracked. I could get a bit paranoid and start leaving my phone at home.” (M001)	C
**Autonomy, freedom, and control**		
Fear of judgment or embarrassment	“Could be doing something with drinks like you’re always down Wetherspoon’s on a Saturday having a drink and then you’re feeling miserable the next day you know.” (C007)	D
	“If I was still taking drugs like I used to in the past I would have said no to [passive sensing] aye but I’m not on drugs anymore so I wouldn’t mind.” (E003)	E
	“I feel like I’m entitled to make my own analysis and to express to my psychiatrist how I’m feeling on a daily basis.” (E001)	F
	“Numbers of text messages sent and received, maybe they’re like oh I don’t want you to know that I’ve been texting [fictional name] I’m in a relationship.” (C007)	G
	“Someone might not be in a good relationship and they’re spending time there … And where could it go anyway … You’re not going to offer domestic violence help … so it could get very tricky.” (K002)	H
	“You might be having sex with someone you know, you wouldn’t want them to be in on that.” (E009)	I
Perceived pressure to change behavior to gain clinician approval	“Imagine somebody’s daily activities are being monitored and then they feel like ‘oh well I need to make a go of it to show the psychiatrist what I want the psychiatrist to think, so I’m gonna get up and I’m gonna increase my steps and I’m gonna meet more people and I’m gonna sleep more and I’m gonna live my life according to the data’. I think that will be a problem.” (E001)	J
	“If its constantly going out to … my keyworker or whatever then I don’t think I’d like it cos I’d feel like I’d have to go out some days, even if I didn’t want to, just so it shows … I had been out.” (G008)	K
	“I maybe only wear my Fitbit if I’m doing exercise, cos I don’t want to have some sort of micromanaging thing … I don’t think I would want to be notified about all the passive information that is being gathered cos most people … don’t want any external information to influence … how they are doing their day-to-day tasks.” (G008)	L
Wearables promote behavior change	“Because I was tracking it, it made me more motivated to increase my steps and get out the house more and just go for walks.” (C001)	M
	“You set a distance … Then at the end of it you get a medal to say you have done it.” (G001)	N
	“I’ll be walking along, or running along, and suddenly my Fitbit would … start vibrating, because I’d done so many steps, and that was quite nice … a little burst of, oh, well done, from my Fitbit.” (K001)	O
	“I have noticed long ago being sedentary is not very good for me. Typically, if I stop exercising, I notice. A lot of people with psychosis, stress is not great and that is one way that can help.” (G001)	P
	“In one mindset … it’s a good thing, you know, there’s loads of benefits to it … but then at the same time … when you’re not in a well mindset … It can kinda do the opposite.” (M012)	Q
**Suggested solutions to address privacy concerns**		
Choosing data types to share with the DRM system and clinicians	“Having options maybe that we can turn this bit off if you’re not comfortable about say your location … we can compromise on that.” (M011)	R
	“If there were filters in place, then I wouldn’t feel worried about it because I’d feel in control of what’s being sent.” (M008)	S
	“I don’t think I would [disable location sharing], because I would like to track … where I am and stuff … but, just to have the power to be able to ….” (G005)	T
	“… separating it into physiological symptoms and … day-to-day personal things like number of calls, texts, messages and location, I think they should be distinguishable, and you should be able to choose which side of the app you want to use.” (M008)	U
	“It’s making me a bit … not feel right, for someone else to get a hold of your data. Doesn’t matter, it’s okay for me to show, but it’s not right for someone to [get hold of it].” (E005)	V
	“If I can choose, sometimes when I feel really unwell, probably I would click yes.” (G009)	W
Temporarily stopping data collection or sharing	“Overall, it is a good idea, but I’m stuck with all the privacy thing that maybe there’s a button you could press on the phone that ceases all activities ‘til you press it back on again. Not just the on/off switch, but a button that you could press that gives you privacy.” (M003)	X
Sharing less precise location data, deductive subtheme	“Actual distance is fine. I know a Fitbit gives you an estimate of that, it is not based on GPS, it is just based on steps … I suppose I am just concerned about battery running out, practicalities.” (G001)	Y
	“My phone is like a safety thing for me. I wouldn’t feel great if it were running out of battery.” (G001)	Z
	“I’m a conspiracy nut … any distance you put into walking speed … you can still work out roughly where someone’s going.” (K008)	AA
Managing ongoing consent for passive sensing	“I’d like to know everything about it. I’d like someone to sit down, like we are now, and talk until … it’s been explained the best way possible for me to understand. And then I’d make a decision. But I’d like it to be objectively not all positive to … the phones and the apps. We’ll do all this with silver bells when it doesn’t tell you that it’s tracking your location and what you’re doing.” (M003)	BB
**Weighing benefits against privacy concerns**		
Cost–benefit analysis, trade-off	“It’s obviously taking a bit of your privacy away, right? If that’s what you’ve gotta do to keep yourself mentally stable then that’s fair enough innit.” (C006)	CC
	“People might think it is an intrusion of their privacy … but … I would feel like I’m being watched over which would be good for me actually.” (M014)	DD
	“I don’t mind a bit of intrusion if it’s gonna help you, I think that’s taking it too far.” (S004)	EE
“I don’t see the point” of passive sensing	“The premise here is that it would help me to observe my activities which would provide an explanation to my emotions. However, I feel like the monitoring of the activities could be done by me. I feel like the explanation of the activities could be done by me.” (E001)	FF
	“I don’t feel as though I’ve got too much going on in my life … that’s problematic so I don’t feel the need for it.” (S004)	GG
	“When I feel really … unwell and keep like locking myself in my room I feel like it is okay [to track location] because I’m not going anywhere (laughs).” (G009)	HH

### Theme 1: Mixed Reactions to Privacy Implications

#### Visceral Reactions to Potential Privacy Violation

Most participants (43/58, 74.1%) acknowledged potential concerns related to privacy. However, their apparent strength of feeling varied. Some (7/58, 12.1%) expressed strong visceral reactions, particularly regarding the volume of data collected, repeatedly stating that this is “too much information” (K002, C004, K006) and feels “scary” (C004), “intrusive” (K002), or “offensive” (G009). On hearing about continuous data collection by wearables and smartphones, one participant expressed apparent shocked disapproval (“Jesus Christ,” K008) and, elsewhere, likened passive sensing to surveillance (“What you are asking me to do is basically wear a wire,” K008). Others expressed similar ideas, such as “It’s like you’re being tagged by the police, like you are on parole” (E001), and considered it “a lot to give away, because now, you don’t have a private life anymore … it’s just taking everything” (M006). Most participants expressing strong negative reactions said nothing would change their mind (“There’s no way you could motivate me to do this” K008), reiterating that it was “too much information” (K002) or involved “invasions of privacy” (K008). Nevertheless, 2 participants suggested that having the ability to opt in/out of the collection of (K006) or sharing of (G009) certain data types would increase their comfort with passive sensing (Theme 3.1). A third (M006) was increasingly enthusiastic as she considered potential benefits (Theme 4.1).

#### Minimal Concerns for Some

Many participants (25/58, 43.1%) expressed minimal concerns about passive sensing. Not everyone explained their lack of concern, but those who did outlined 4 reasons. First, some found passive sensing acceptable provided the data would be used for “the right purposes” (C005) or “a good reason” (C001). Accordingly, understanding *why* passive data are gathered seemed a necessary (though not sufficient) condition of accepting the method (see Theme 4). Second, some participants considered the proposed passively collected data types “not too personal” (E004), “nothing that is going to harm a person” (G010), and “[not] a risk to my personal safety or identity” (K003). While not all participants shared this view, these findings highlight that perceived risks or potential harms associated with passive sensing data are important factors influencing participants’ acceptance of such technologies. Third, some participants were unconcerned about a device passively collecting data, viewing it as an expected function of modern technology (“that’s what it does,” K010). They noted that younger generations, as digital natives, were accustomed to this level of data collection (“My generation from I suppose mid 90s onwards have grown up with … being fully aware that the device is in your pocket and what you wear are collecting lots of data on you,” C005). Similarly, participants pointed out the widespread use of location data by other apps: “I think all the apps know where you are so … why *that* app would concern people?” (G005). Fourth, several participants found passive sensing unconcerning because they did not see themselves as engaging in activities that could be scrutinized or misinterpreted (“I’ve got nothing to hide,” E003) and considered themselves “pretty, squeaky clean … I don’t think there’s … anything for me to be worried about” (G002). They acknowledged that others may find passive sensing intrusive (especially location sharing) but, for them, personally, the potential mental health benefits outweighed their minimal privacy concerns (Theme 4.1).

#### Uneasy Feelings About Location Tracking

Many participants (20/58, 34.5%), including some who found passive sensing acceptable overall, described uneasiness about location tracking specifically, feeling “wary” (S004) or “uncomfortable” (M010, K004), or stating that ongoing location monitoring “gives me a bad feeling” (S004), is “a bit worrying” (K007) or “big brother-y” (S004, S007, M011). It was clear from the volume of comments and apparent strength of feeling that participants found location data the most concerning of the data types that might be collected passively. This discomfort was rooted in the fundamental belief that people have a right to privacy: “I just feel like humans, we don’t need everything tracked” (K004). Similarly, participants considered their everyday activities “no one else’s business” (K002), often emphasizing their need for autonomy: “I’ve got choice about where I go … I shouldn’t have to be monitored” (S004); “I want to be able to go places and nobody knows where I am” (E001).

Some participants expressed that, although they had nothing specific to hide, sharing exact location still felt like a privacy infringement: “Not that I’m going anywhere that I shouldn’t be. Ahaha … I just don’t like the idea of somebody being able to say oh she’s in Tescos, y’know” (S007). Unlike participants who expressed minimal concern about passive sensing because of the belief that they have nothing to hide, these participants questioned the value of sharing exact location data, as they were not convinced it offered clear mental health benefits. Views expressed elsewhere indicated that 2 of these participants found sharing other passive data types, including less precise location data, more acceptable than exact location (Theme 3.4) and 2 found it easier to see clear benefits of passive sensing for physical health than mental health: “I know some diabetes, they monitor the sugars … that’s quite different. But mental health is another thing” (M006).

Some unease about location tracking related to participants’ experiences of psychosis or receiving care from services. Several participants cautioned that location sharing may be particularly problematic for people with existing anxiety or paranoia, especially those who were “technology paranoid” (C002), and may lead to feelings of disempowerment and potentially exacerbate symptoms (Table 2, quotes A-C). Participants’ reactions highlighted that the idea of location data being shared with clinicians is not a case of neutral data being shared with a neutral recipient. Rather, it is embedded within complex relational and power dynamics.

### Theme 2. Autonomy, Freedom, and Control

#### Fear of Judgment or Embarrassment

In contrast to participants who considered themselves “squeaky clean,” several participants were strongly against passive data being shared “with a clinical team that might analyze it negatively” (K002). They feared the team would make “judgments based on that type of information” (E001), inferring information about sleep disruption, substance use, and relationship-related activities. While most participants viewed sleep tracking positively, as it could help them recognize and manage sleep difficulties (Theme 4.3), some implied that sharing their sleep data might make them feel judged because “I don’t have a set sleep pattern” (K008) or “my pattern’s all over the place” (M009), with one participant joking they might get “told off for sleeping too much” (C003). These participants appeared to want to improve their sleep before sharing sleep data. Although sleep data was less contentious than other activities that participants highlighted, this illustrates the discomfort some may feel about having their daily activities scrutinized.

Participants anticipated that clinicians would “frown upon” (E009) evidence of substance use, which they thought location data would reveal by identifying them as being in the pub or visiting a drug dealer (quotes D-E). As illustrated by the second quote, former drug-using participants were open to passive sensing, but would have been unwilling to share such data with clinicians while still actively using substances. Participant E009 admitted they would “maybe deceive them or … not let them in on everything”. There was a strong sense that some participants wanted to feel in control of the narrative presented to clinicians, particularly when they felt their actions may be viewed negatively (quotes F).

Regarding relationships, participants suggested that passively sensed location and/or phone/text data may cause unintended disclosure of highly sensitive personal information. Participants worried that location and communication data could expose aspects of their private lives that they may not wish to share with clinicians, such as infidelity (quote G), involvement in abusive relationships (quote H), or information about sexual activity (quote I).

As well as highlighting aspects of individuals’ relationships that may invite judgment or require intervention, passive data could reveal information outside the scope of that usually shared between people with psychosis and clinicians (eg, information about sexual activity). In evaluating passive sensing’s acceptability, one must consider what unintended information (and associated meaning) such data may reveal, particularly when multiple data types are combined and when, in future, algorithms for identifying activities from passively sensed data become more sophisticated.

#### Perceived Pressure to Change Behavior to Gain Clinician Approval

Several participants worried that passive sensing would make them feel under pressure to modify their behavior, ultimately compromising their autonomy, constituting “a curtailment of my personal freedom” (E001) and “making more of a subject of the patient and less of a person” (E001). Participant E001 emphasized this multiple times, giving examples of how people might change their behavior to act in ways they think clinicians will approve of, forgoing their right to make their own choices about their needs or preferences. As quote J emphasizes, passive sensing may prompt someone to make positive changes, but doing so at the expense of their autonomy is likely to be problematic.

Even participants who considered their current choices were the most suitable for their personal circumstances felt that sharing passive data with clinicians might make them feel like they “have to” change their behavior. For example, participant G008 worked from home and sometimes preferred not to go out after work (quote K).

Participants’ instinct to demonstrate certain “healthy” behaviors to clinicians may stem from previous encounters with a paternalistic healthcare system, where professionals are seen to know best and people using mental health services can feel infantilized. In this context, there is a danger that sharing passive sensing data with clinicians may reinforce and perpetuate pre-existing power imbalances.

#### Wearables Promote Behavior Change

Some participants warned that people can end up “fixating on” the data (C002), “having an obsessive sort of unhealthy relationship with how many steps you’ve done or how many calories you’ve burned” (M010), and that it “contributes to a bit of health anxiety” (C002) and may be particularly unhelpful for people with eating disorders. Similarly, another participant (quote L) expressed feeling pressured by devices to adjust their behavior and preferred not to receive a copy of passive sensing data (see also Supplementary Material S7). Nevertheless, others (quotes M-O) described using devices to support their health goals and increase their motivation to make positive physical and mental health changes.

Features designed to promote behavior change may be viewed differently by individuals who are internally motivated to make lifestyle changes (eg, quote P describes clear intrinsic motivation to exercise), compared to those who feel compelled to change due to perceived disapproval from clinicians (external motivation) or devices. Nevertheless, having a more complex relationship with exercise may result in mixed views about using wearables for passive sensing. One participant described past difficulties using a Fitbit (“I got obsessed with the gym and calories and stuff … so I had to stop using it,” M012) but also acknowledged that reviewing passively sensed data could promote healthy choices (“It … motives you like oh I’ve not been out in a few days, I should probably go out,” M012). She hypothesized that her experience would vary depending on her mental health (quote Q).

### Theme 3: Suggested Solutions to Address Privacy Concerns

#### Choosing Data Types to Share with the DRM System and Clinicians

Several participants spontaneously suggested that users should be allowed to choose which data types they will share with the DRM system (quotes R-T), with location most often mentioned. Even participants who were willing to share all data types expressed a preference for having the option to choose (quote T). The use of words like “power” and “control” underlines that having a choice about what data to share is empowering for users and essential to maintaining a sense of autonomy. Conversely, not having a choice may be disempowering, mirroring participants’ concerns about their privacy (Theme 2.1) and autonomy (Theme 2.2) being undermined by sharing unfiltered passive sensing data with clinicians.

Acknowledging that data types varied in acceptability, M008 suggested grouping these into *health data* and *personal data* and giving DRM users a choice about sharing data from these categories (quote U). The same participant alluded to 2 ways of allowing users to opt in/out of sharing data: being able to choose “the types of information that are collected” or “being able to filter the information that’s sent” (M008). The first option would stop the DRM system from collecting specific data (eg, location) from users’ devices altogether. The second option would allow users’ devices to share data with the system but to prevent the system from sending certain data types to clinicians. Other interviewees did not distinguish between these concepts when advocating user choice over sharing specific data types. However, for most participants, the core difficulty with passive sensing was the feeling that their privacy was invaded if other people (especially clinicians) could see their data (especially location data). Therefore, giving users the option to allow the DRM system to gather passive data without transmitting all data types to clinicians may be an acceptable compromise.

Some participants who opposed real-time sharing of all passive sensing data types with clinicians were still open to discussing and sharing this information during in-person appointments. They contrasted the intrusiveness of automatic, real-time data sharing (“stealing information,” E005) with having control over sharing it themselves. Again, there was a clear emphasis on users preferring choice about data sharing and wanting to feel in control of the narrative presented to clinicians (quote V).

Sharing data only in person would negate some key advantages of DRM (remote, real-time use). As a potential compromise, one participant who felt uncomfortable with continuous passive sensing suggested a DRM system feature that would allow clinicians to request specific data remotely on a time-limited basis. This approach would enable users to decide in the moment whether they consent to sharing the requested information (quote W).

#### Temporarily Stopping Data Collection or Sharing

Some participants who were generally open to real-time data sharing wanted the option to stop sharing data (M003), particularly location, at certain times (eg, during “sensitive medical appointments,” M003). Participant C002 reflected on their routine wearable use, explaining that they choose, daily, whether to wear their smartwatch, based on current paranoia levels or simply preferring not to wear it sometimes. They acknowledged this pattern may not provide an ideal input for a DRM system’s relapse prediction algorithm (“for it to work, I think it would have to be quite consistent with the use,” C002) and suggested the system could let users specify, daily, whether they will use the wearable. They emphasized that this would support user independence and autonomy while guarding against potential inaccuracy.

#### Sharing Less Precise Location Data

Participants’ preferences about location precision varied. Half of participants who commented on this preferred the DRM system to gather exact location data, primarily because it was considered helpful in an emergency (see Theme 4.3). Half preferred non-exact location, explaining that it “feels less intrusive” (S005) than exact location, prompts less perceived pressure to act differently and fewer privacy-related worries about being followed or monitored. It may also deplete their device’s battery less, which was a key consideration for one participant (quotes Y-Z). Nevertheless, one participant worried that even non-exact location measures (distance traveled from home) would reveal their actual location (quote AA).

#### Managing Consent for Passive Sensing

Participants underscored that fully informed consent is crucial prior to using passive sensing methods and several concluded that passive sensing is acceptable if informed consent is granted. For instance, once prospective users had been given as much information as possible, “from there I suppose it’s up to the individual” (C005). A full and balanced explanation was emphasized (quote BB). We asked interviewees how often they would choose to reconfirm consent for passive sensing; monthly consent was the most frequently preferred option among responders (see Supplementary Material S8).

### Theme 4: Weighing Benefits Against Privacy Concerns

#### Cost–Benefit Analysis (Trade-Off)

Participants often directly weighed their privacy concerns against the system’s usefulness. Some concluded that, on balance, potential perceived benefits, such as staying well (C006) or feeling “watched over” by clinicians (M014), outweighed the privacy concerns (quotes CC-DD). Others concluded that, for them, the added value of passive sensing was not worth the privacy sacrifice (quote EE). The process of weighing the privacy cost against specific benefits is highly individualized. As one participant emphasized, “each person has to weigh it up for themselves and figure out what’s more important for them” (K004).

Findings presented in Themes 1-3 show that individuals’ perceptions of the *costs* of using passive sensing within a DRM system are shaped by diverse personal and contextual factors. Similarly, perceived *benefits* also vary widely. Participants’ views sometimes shifted as the interview progressed, suggesting that the perceived balance of costs and benefits can evolve as individuals consider more information, as noted in the author EE's analysis diary:

*“Participant’s opinions seem to develop as the interview progresses. Initially she is hesitant about providing lots of passive data (‘it’s a lot to give away’) and would only do so if very well financially reimbursed. Later, though, she says ‘it’s fine because you have a lot to gain’, which contrasts with her opinion that it’s a lot of data to give away.” (analysis diary notes on M006 interview)*

Although initially opposed to passive sensing due to perceived privacy costs, this participant’s overall view of the DRM system adjusted as she considered its potential benefits.

#### “I Don’t See the Point” of Passive Sensing

Not all participants thought the potential benefits of passive sensing outweighed the privacy costs. The view that they themselves are best positioned to interpret their behaviors and experiences reflects a broader desire for self-sufficiency and autonomy in managing mental health data (quote FF). One participant, who acknowledged the potential benefits for others, felt that these benefits were not personally compelling or relevant at the present time (quote GG). Similarly, others noted that passive sensing’s relative costs and benefits might fluctuate over time (quote HH). When feeling well and maintaining their usual routine, they perceived a higher privacy cost and less benefit; conversely, when unwell and staying at home, the sense of privacy intrusion felt less acute, and benefits more apparent.

#### Specific Benefits of Passive Sensing

Participants outlined 4 specific benefits of using passive sensing within a DRM system (Table 3): ease of use, value in identifying possible relapses, benefits of sleep monitoring, and utility of sharing precise location in an emergency.

**Table 3:** Specific Benefits of Passive Sensing Outlined by Participants

**Benefit**	**Summary of main point(s)**	**Supporting quotes (main points)**	**Summary of counterpoint(s)**	**Supporting quotes (counterpoints)**
Ease	The ease of passive sensing was contrasted with the burden of answering questions via ASM. This ease is particularly beneficial during illness episodes: even if they did not feel “up to proactively doing something” (M008), individuals’ mental health is still monitored.	“You don’t have to plug in specific numbers into a phone, it just sort of does it for you.” (M010) “The passive way is better … ‘cause I remember when I was at my worst, I don’t know if I would have been able to engage with my phone.” (K007)	Although acknowledging passive sensing’s ease and convenience, one participant warned that privacy impacts still need considering and may be especially unsettling when participants are unwell (eg, when experiencing paranoia).	“I think for memory or for convenience it’s good, but … relating to psychosis, that could be scary for someone and it might even push them away a bit more because they know that their phone is capable of doing that.” (C004)
Flagging possible relapses	Several participants recognized that anomalies in location or movement tracking (eg, over-activity/under-activity) could flag possible relapses. They gave examples from their own experiences. Participants noted that they sometimes avoid seeking help so as not to cause worry but that receiving a call from a clinician (prompted by passive sensing data) could be: “really engaging and really useful.” (S004)	“Moving around possibly too much … getting up in the middle of the night or … going into places where you shouldn’t be going … or the other extreme just not going out at all.” (S004) “When we suffer with mental health we close ourselves in. And we don’t wanna inform other people ‘cus we don’t want them to worry. But then at the same time, we’re isolating ourselves and we’re not interacting with anyone, so yeah that is good with the location.” (C007)	Participants cautioned that decontextualized passive data may cause false positives (flagging a relapse when someone is well) and that differences in circumstances (eg, low baseline activity) or activities could influence passive sensing data in ways not directly related to mental health. One participant suggested that DRM users could provide context to help staff interpret future passive sensing information. They suggested that this may have the added advantage that it “probably won’t feel as if you’re stealing from them.” (C005)	“I think it’s a good idea, but it would do nowt with me ‘cause I only go out for about ten minutes a day.” (M009) “Really depends on … how much you have to do and where. If you’ve got any other outdoor resources like … mental health or workplace or voluntary or whatever.” (E006) “A lot of that information will probably come in quantitative form … a lot of numbers … whereas I think … practitioners using the app would benefit from maybe getting a qualitative understanding … a summary of someone’s health … before the app is installed.” (C005)
Sleep monitoring	Overall, participants were positive about sleep tracking, considering it “interesting” (E004), and useful for recognizing links between sleep and mental health, assessing severity of sleep difficulties, and taking steps to improve these.		Not everyone was convinced the benefit outweighed privacy costs. Some questioned the accuracy of the sleep data or considered it “quite personal to gather.” (K009)	“I do have mixed feelings. I don’t think I’m completely decided.” (E001) “It is not polysomnography, it is not super accurate the sleep thing, but it gives you a bit of an indicator.” (G001)
Precise location data helpful in an emergency	Participants who preferred exact location to be gathered considered this useful for getting help in case of accident (“If I fell somewhere,” E003), medical emergency (“having a heart attack,” G010), or mental health crisis (“people with suicidal thoughts,” S005). Several participants mentioned occasions they had gone missing for mental health related reasons (eg, command hallucinations or dissociation). As these participants highlight, location data could provide reassurance to family members and/or help individuals reconstruct their own actions when dissociated.	“The more precise it is the better probably.” (E007) “I’d feel safer … if I do need any help.” (M014) “[Hallucinations] would tell me where to drive and go, so I was going all random locations. It would have been good for my parents to like, ‘hang on now he’s down [location] how the heck did he get down there (laughs) all of a sudden? He’s in a psychotic episode’.” (C007) “I had a dissociative episode and lost like 7 hours and because of the app, I was able to track where I had been, which was really useful.” (S008)	Others acknowledged occasional advantages but would not agree to gather continuous location data.	“It’s helpful … say you got lost … But other times … you’re going about your usual day and it’s tracking you, I don’t really like that.” (M012)

## Discussion

This study examined participants’ views about using passive sensing within a DRM system for predicting psychosis relapse. With its large, diverse sample, detailed topic guide and extensive lived experience involvement, it is the most in-depth study to examine the views of people with psychosis on this topic to date. Participants expressed polarized feelings about passive sensing and highlighted specific benefits and challenges. Privacy was a key concern: participants worried that sharing passive data (especially location) may reveal sensitive information and impact autonomy. Offering choice about what data are shared, when, and with whom may help address privacy concerns and increase engagement. Our findings underline the importance of fully informed consent: prospective users of a DRM system need clear, accessible information about how passive data will be collected and used, and about relevant costs and benefits so that they can make an autonomous and informed choice. As well as recognizing and valuing some of the intended functions of passive sensing within DRM (ease of use, early identification of relapse, relevance of sleep monitoring for mental health), participants highlighted novel uses of passive sensing data (using location data to summon help in an emergency or crisis). While some questioned the value of passive sensing, others thought its benefits outweighed privacy concerns. Below, we compare our findings to existing empirical and theoretical literature and provide recommendations.

Studies reporting the views of people with psychosis about DRM often mention privacy, but usually in general terms alongside security worries about ASM-generated symptom information being disclosed to third parties.^17^ Our findings suggest that people with psychosis express more intense and specific privacy concerns about passive sensing than ASM. The volume and type of data collected, and whether data are shared with clinicians, were important privacy-related factors. Despite no directly analogous studies, the wider digital health literature yields comparable findings.^36^^,^^49–55^ Participants’ misgivings about the volume of data collected echo clinicians’ concerns that accessing “vast amounts of information” via passive sensing invades users’ privacy.^36^ Similarly, our finding that participants were more willing to share health information (eg, heart rate, sleep data) with clinicians than personal information (eg, location, number of calls/texts), mirrors findings from studies with the general population,^49–51^ adolescents with mental health problems,^52^ and people with psychosis.^56^ Others have explained this distinction using the Contextual Integrity Framework: sharing health data with clinicians complies with existing information norms, whereas sharing personal data does not.^51^^,^^57^^,^^58^

Regarding the data recipient’s role, general population participants and people with depression prefer sharing passive sensing data with clinicians than non-clinicians.^49^^,^^51^^,^^55^ Conversely, some participants with psychosis were uncomfortable sharing passive sensing data with clinicians, anticipating negative consequences and reduced autonomy. This disparity may arise from different experiences of patient-clinician relationships in respective study populations. There is typically an inherent power imbalance in secondary care mental health services.^59^^,^^60^ Clinicians are positioned as “experts” and, under certain circumstances, are legally empowered to restrict patients’ freedoms (eg, under the Mental Health Act^61^). Individuals who have experienced paternalistic or coercive care may be particularly cautious about sharing passively sensed data; such experiences can shape perceptions of autonomy and influence their willingness to engage with DRM systems.

As well as potentially exacerbating existing power imbalances, passive sensing may further undermine users’ autonomy if they are not given ongoing choice about usage. Self-determination theory posits that autonomy, competence, and relatedness are central to motivation and engagement.^62^ In the context of passive sensing, self-determination is limited but not entirely absent. The initial decision to wear a device (eg, smartwatch) reflects an element of autonomy: users choose to engage with the technology. However, once the device is worn, passive sensing operates unobtrusively, removing ongoing opportunities for autonomy, as users do not actively decide when or how data are collected. Unlike ASM, where individuals directly provide data, exerting agency over monitoring, passive methods minimize user involvement. This raises an important theoretical challenge: if autonomy drives motivation, does the absence of ongoing autonomy in passive sensing impact engagement, adherence, and outcomes? While passive sensing reduces burden and increases objectivity, it may also limit perceived control over one’s data, potentially influencing acceptance and long-term use. Future work should explore whether and how elements of self-determination theory can be integrated into passive sensing frameworks, perhaps through hybrid models incorporating moments of user reflection or choice, to optimize both autonomy and utility. Our findings suggest that allowing users choice (initially and over time) about the types of data collected/shared may provide agency over passive sensing data and address privacy concerns. Similarly, monthly renewal of consent for passive sensing appeared suitable; future research should explore this finding quantitatively.

### Recommendations

Given the potential impacts on privacy and autonomy, clinicians or researchers introducing a passive sensing-based DRM system to people with psychosis must manage informed consent thoroughly. As with other health interventions (eg, medication), we recommend setting aside time for an in-depth conversation with prospective DRM users to discuss benefits and side effects. Table 4 outlines specific, detailed recommendations arising from our findings. We will highlight 3 key points. First, descriptions of passive sensing must be transparent, and clear to users. As Mohr and colleagues argue,^63^ medicalized terms like “digital phenotyping” may imply that passive sensing is inherently scientific, accurate, necessary, and too complex for users, potentially obscuring privacy implications of passive sensing and discouraging critical engagement with investigating the method further. To combat this, we recommend collaborating with people with relevant lived experience to develop clear, lay descriptions of passive sensing and to agree on appropriate terminology. Second, as with the general population,^50^ people with psychosis need to see a benefit of passive sensing for themselves personally (not just for clinicians) or they are unlikely to use it outside of a research context (where altruism may be a key motivation). The current qualitative study outlines benefits from participants’ perspectives and highlights that users may consider opting into passive sensing for unexpected reasons (eg, feeling reassured by sharing location). Similarly, EMPOWER study participants engaged with an ASM system because it afforded benefits that were personally meaningful to them (eg, social connection); the authors described this with reference to the well-established “affordances” literature.^64^^,^^65^ Third, as well as outlining potential harms to prospective users, researchers/clinicians should monitor and report passive monitoring’s adverse effects.^66^ As empirical data on adverse effects of passive sensing are scarce,^67^ it is important to watch for a wide range of potential harms; eg, our participants hypothesized that using wearables may exacerbate co-morbid eating disorder symptoms. Our participants also worried about passive sensing revealing unintended personal information (eg, about sexual activity). If passive sensing is used in health services, it will be vital to consider not only whether such unintended information contravenes social norms about data sharing but also whether it may cause direct or indirect harm to individuals. Potential harms (eg, revealing information about someone’s sexual activity) to individuals from marginalized communities (eg, sexual minorities) will be particularly important to consider, with reference to the wider socio-political context, time, and place in which the technology is used (eg, a country or subculture where there is reputational or legal risk to information about one’s sexuality being revealed).

**Table 4:** Recommendations for Clinicians and Researchers Planning to Use a Passive-Sensing-Facilitated DRM System With People With Psychosis

**Findings from the current qualitative study**	**Recommendations for consent or onboarding session(s)**		**Design or *research* ideas**
**Overall consent process**			
Fully informed consent to use passive sensing is crucial.	Provide a clear, transparent explanation in lay language covering: how the system works, what data is collected, how data stored and used, potential costs/risks, and potential benefits. Consider co-designing a script with people with lived experience of psychosis to ensure clear explanation of all relevant information.		In-app information section for users to refer to in their own time and when re-consenting.
The process of weighing the costs and benefits of passive sensing is highly individualized and can evolve over time, especially as additional information becomes available.	Encourage individuals to ask questions to obtain extra information about aspects of the system that are most interesting or concerning for them, personally. Allow time for people to consider the information on their own (or with trusted others) and provide opportunities to ask further questions before deciding.		
Some participants had strong negative reactions to passive sensing and could think of nothing that would increase their comfort with the idea	Be aware that not everyone will want to use passive sensing. If used in healthcare, it should be presented as an option alongside other mental health tools, but without pressure to comply. Allow individuals to make a free choice.		
Individuals’ willingness to use passive sensing is likely to vary over time. It may fluctuate in line with their mental health.	Consider re-confirming consent for passive sensing periodically. Consider co-creating an advanced directive with DRM users stating whether or not they wish to continue to use passive sensing if they lose capacity to consent.		Option to stop data sharing (short/long term).
Preferences for consent renewal frequency varied but monthly was the most preferred option.	Monthly renewal of consent may be suitable. If possible, allow individuals to specify their preferred frequency.		Monthly in-app message. *Survey of a large sample.*
**Explaining how passive sensing works and its potential benefits**			
Participants were more willing to use passive sensing if they thought data was collected for a good reason.	Explain specific reasons for collecting each data type clearly.		Ensure no redundant data is collected.
Compared to ASM, the link between passive monitoring and mental health changes was harder to understand.	Be aware that the link between passive monitoring data and mental health may not be intuitive. Ensure that attention is given to explaining the link during consent/onboarding.		
Understanding the benefits of passive sensing was important. Participants said they would need to see a benefit to themselves, personally, or they would not use it. Some unexpected benefits of passive sensing were highlighted (eg, using location data in an emergency).	Be aware that prospective users’ reasons for agreeing to passive sensing may not match clinicians’ reasons. Explain all potential benefits. For example, passive sensing can: be lower burden than ASM for users; flag mental health deteriorations; send information to clinicians when the person with psychosis does not feel well enough to do so; provide location data in emergency or crisis; reassure family members (or nominated trusted other); facilitate sleep tracking.		
The use of concrete examples helped participants to understand how passive sensing might help to identify changes in mental health.	Use concrete examples to explain how passively sensed data can help identify mental health changes. eg, “If someone starts feeling very anxious or low, they might start socializing less. Information gathered from their smartphone might show that they are staying at home more and messaging people less. This could help us spot that their mental health is getting worse and provide extra support.”		
Some found it easier to see clear benefits of passive sensing for physical health than for mental health.	Consider using physical health monitoring as an analogy to explain how passive sensing can help track mental health. If potential users would also benefit monitoring their physical health with a wearable (alongside mental health), provide support for this.		
Participants were interested in sleep and considered it relevant, although some considered it quite personal. Participants were curious about the impacts of poor sleep on mental health and wanted support to manage it.	Discuss sleep data with interested individuals and provide (or refer for) appropriate support. As with other data, individuals should decide whether sleep monitoring benefits outweigh the privacy costs for them, personally. The key task for clinicians/researchers is to give clear information about possible benefits.		
Some participants warned that using consumer wearables (eg, FitBit) to monitor sleep may not be as accurate as gold standard devices.	Provide data on sleep monitoring accuracy for specific devices. Advise using wearables as a relative (not absolute) measure of sleep, ie, to identify sleep *changes*. Advise that, for people with sleep disorders, wearables may be consistently inaccurate in measuring sleep but that other data (eg, activity level) is still useful for monitoring mental health.		
Given that passive sensing data lack context, some participants questioned its accuracy in identifying relapses, suggesting it may cause misunderstandings.	Provide data on accuracy, sensitivity and specificity. Ensure clinicians and DRM users understand the limitations of passive sensing for identifying mental health deteriorations. Reassure DRM users that the clinical team are aware of the system’s limitations and will interpret the data accordingly.		*Collect data on accuracy of passive sensing data for predicting relapses.* *Explore stakeholder views on suitable balance of sensitivity vs. specificity.*
**Understanding privacy implications and other potential harms**			
Perceived risks or harms of passive sensing data were important factors influencing acceptance.	Explain possible risks/harms. Check if the individual perceives specific personal risks or harms from data types collected. Provide further information on these if needed.		*Report data on actual harms caused by passive sensing.*
Participants were worried about passive sensing revealing unintended personal information (eg, about sexual activity).	Consider whether unintended information contravenes social norms about data sharing and whether it may cause direct or indirect harm to individuals. Pay particular attention to potential harms to individuals from marginalized communities, with reference to the wider socio-political context, time, and place in which the technology is used.		
Some participants were strongly against passive data being shared with their clinical team.	Some people may be willing to share some/all data types with a DRM system but not with the clinical team. Explain what options the specific DRM system you are using allows, such as: (i) sharing data with the DRM system for self-monitoring only (no data or alerts shared with the clinical team); (ii) sharing data with the DRM system and sharing relapse alerts with the clinical team (detailed data not shared); and (iii) sharing detailed data and relapse alerts with the clinical team.		Allow users to specify whether data is shared.
Data type was important to participants, with certain data types considered more private, especially location data and number of calls/texts. Participants were less willing to share personal than health data.	Discuss with prospective DRM users which data type(s) they were willing for the DRM system to (a) gather and (b) share with the clinical team. For ease of understanding, consider grouping data types into *health data* and *personal data* and give DRM users a choice about whether the system gathers and shares data from these categories. Provide information on whether opting out of collecting certain data types affects the DRM system’s accuracy in identifying relapses.		Allow users to specify which data types are (a) collected and (b) shared.
Some participants wanted to feel in control of the narrative presented to clinicians. They would not agree to ongoing passive monitoring but may be willing to share time-limited passively sensed data on request.	Explain clearly how the data will be used and what information will be conveyed to the clinical team. Ensure that the individual feels comfortable with this before using the DRM system.		Add a feature whereby clinicians can request, or participants can send, specific data on a time-limited basis.
Some participants found passive sensing unconcerning because they felt they had “nothing to hide” from the clinical team.	Check privacy implications are fully understood before proceeding. This view may reflect a broader assumption that passive sensing is only problematic when there is something to conceal, potentially overlooking broader implications of continuous data collection.		
**Considering individual characteristics and contextual factors**			
DRM systems and passive sensing may be especially concerning for those with existing anxiety/paranoia about technology.	Consider the person’s clinical history when deciding whether to use DRM as part of their care plan.		
Location sharing may also be especially concerning for those with existing anxiety or paranoia about surveillance or being followed, whereas others saw it as a potential benefit in an emergency.	Clearly explain what data is being collected (exact location, general area, distance traveled, etc.) to help the individual decide whether this would be acceptable to them.		
One participant worried that gathering non-exact location data (distance traveled from home) would allow their exact location to be inferred.	DRM users may need reassurance or further information (eg, about what can be inferred from non-exact location data) to assuage specific concerns		
For people who rely on their phone (eg, for safety), the extent to which passive sensing drains their phone’s battery may be a key consideration.	Ask about prospective users’ practical concerns about using the DRM system. Provide information to help them decide whether using the system is practical for them. For example, the extent to which passive sensing will drain their phone’s battery.		
Our findings highlighted that sharing passive sensing data with clinicians may reinforce pre-existing power imbalances in some patient-clinician relationships.	Consider the specific context of the individual’s relationship with their clinical team and how using a DRM system may impact this relationship. Ask prospective users if they expect passive sensing to reduce their autonomy and/or give the team more power.		
We found no clear pattern of demographics (eg, age) or UK geographical location on willingness to use passive sensing.	Do not restrict DRM to certain demographic groups. Reflecting findings in the general population (Nicholas et al., 2019. Klansnja et al., 2009), demographic characteristics (eg, age) did not appear to influence willingness to use passive sensing.		*Quantitatively examine the impact of demographic factors in a larger sample.*
Individuals who are accustomed to smartphone and wearables gathering ongoing data may find passive sensing more acceptable.	Familiarity with phones/wearables gathering data may influence acceptance of passive sensing. Ask about previous experience/familiarity with this. For those unfamiliar with these technologies, consider offering DRM (and/or a smartphone/wearable) for a trial period to gain familiarity before deciding.		
Wearables promote behavior change. People internally motivated to make lifestyle changes may view this differently than those who feel compelled to change by clinicians or devices (external motivation).	Be aware that wearables promote behavior change; for some this can be positive but for others it may be anxiety provoking or perceived as unduly pressuring. Address this directly in an expectation-setting conversation to ensure users do not feel undue pressure to change their behavior but feel supported to make healthy changes that they decide on themselves.		
Some participants expressed a preference for exact location data to be available in the event of a crisis.	Using exact location data, within clinical practice, to locate users in the event of a crisis would require thorough consideration prior to implementation due to the obvious practical and ethical implications. Clear expectations regarding clinical workflows (eg, would location data be monitored out of hours, and if so, by whom?) and responsibilities for responding to such data (how soon should a response occur? What should happen when the individual has been located?) would need to be established. Consultation with relevant stakeholders (eg, clinicians, people with psychosis) would be important to identify further unforeseen consequences and concerns and to establish whether there are any circumstances in which such a system would be practical and valued by users.	*Seek clinician views on the acceptability of using exact location data to find DRM users during a crisis.*	

ASM = active symptom monitoring; DRM = digital remote monitoring; UK = United Kingdom

### Strengths and Limitations

This study had important strengths. We recruited a large, diverse sample from 9 NHS Trusts/Health Boards across 6 geographically distinct urban and rural areas of the UK. We involved lived experience contributors extensively throughout the study, to ensure information, questions, and language were accessible and appropriate, and that analysis remained grounded and relevant in the real-world. We employed an interview technique whereby we gave participants an initial explanation outlining the concept of passive sensing before using flexible, open questions to elicit their views. This allowed us to gather rich data exploring participants’ perspectives on a complex, unfamiliar concept, and to explore their initial views and how these developed over time during the interview. As others have observed,^68^^,^^69^ within qualitative interviews, participants’ views may shift as they engage in deeper reflection, encounter new perspectives, or consider aspects they had not previously thought about. The interviewer’s probing questions may encourage them to articulate and critically examine their own thoughts. In addition, discussing benefits, risks, or alternative viewpoints may prompt reconsideration or refinement of their stance. There were also some limitations. Although we used purposive sampling to gather diverse perspectives, we only interviewed English-speaking participants. Similarly, while our sample included a range of experiences of digital health, people with more interest in DRM may have been more likely to participate. Therefore, participants’ views may not be representative of the broader population of people with psychosis.

## Conclusions

Our findings suggest that, although passive sensing may be acceptable for many people with psychosis, unless carefully designed and managed, it may cause distress, fear of judgment, or reluctance to engage with DRM, particularly for individuals navigating complex personal circumstances. This underscores the importance of clear consent processes, data transparency, co-design of digital systems with stakeholders, and user control over what information is shared and how it is interpreted by clinicians.

## Supplementary Material

Connect_SU_quali_Paper_1_-_supplementary_material_02_05_25_sbaf126
