# Supplementary material for: Using Passive Sensing to Predict Psychosis Relapse: An In-Depth Qualitative Study Exploring Perspectives of People With Psychosis
Source: Schizophr Bull. 2025 Aug 21;52(4):sbaf126. doi: 10.1093/schbul/sbaf126 (PMC13391645; doi:10.1093/schbul/sbaf126)

**Using passive sensing to predict psychosis relapse: an in-depth qualitative study exploring perspectives of people with psychosis**

**Supplementary material**

Contents

[S1. Consolidated criteria for reporting qualitative studies checklist 2](#_Toc197100773)

[S2. Topic guide 7](#_Toc197100774)

[S3. Supplementary methods 19](#_Toc197100775)

[S4. Health apps installed on participant devices 20](#_Toc197100776)

[S5. Supporting quotes per theme 21](#_Toc197100777)

[S6. Summary of views on specific passive sensing data types 32](#_Toc197100778)

[S7. Answers to the question “Would you want to see a copy of the passive sensing data that the system collects?” 36](#_Toc197100779)

[S8. Participants’ preferences regarding frequency of passive sensing consent renewal 38](#_Toc197100780)

###

### S1. Consolidated criteria for reporting qualitative studies checklist

| **No** | **Item** | **Guide questions/description** | **Notes on relevant content** | **Page of manuscript** |
| --- | --- | --- | --- | --- |
|  | **Domain 1: Research team and reflexivity** | |  |  |
| 1. | Interviewer/facilitator | Which author/s conducted the interview or focus group? | Cara Richardson, PhD  Hannah Ball, DClin  Kathryn O'Hare, MSc  Laura Maclean, PhD  Natalie Chalmers, PhD  Rebecca Turner, PhD  Sybil Clifford, MSc  Sophie Faulkner, PhD  Uzma Zahid, PhD | Supplementary materials S3, reflexivity |
| 2. | Credentials | What were the researcher's credentials? *E.g. PhD, MD* | All had a PhD, DClin or MSc | Supplementary materials S3 |
| 3. | Occupation | What was their occupation at the time of the study? | Researchers on the CONNECT study | Supplementary materials S3 |
| 4. | Gender | Was the researcher male or female? | All female | Supplementary materials S3 |
| 5. | Experience and training | What experience or training did the researcher have? | Relevant degrees and CONNECT training | Supplementary materials S3 |
| 6. | Relationship established | Was a relationship established prior to study commencement? | Researcher not usually known to the participant | Supplementary materials S3 |
| 7. | Participant knowledge of the interviewer | What did the participants know about the researcher? e*.g. personal goals, reasons for doing the research* | Not known aside from info presented in the participant information sheet | Supplementary materials S3 |
| 8. | Interviewer characteristics | What characteristics were reported about the interviewer/facilitator? e.g. *Bias, assumptions, reasons and interests in the research topic* | “All researchers who conducted interviews or analysis had some background knowledge of the topic area and may have brought their own assumptions and biases. For example, they may have felt invested in the idea of a DRM system that includes passive sensing being used within mental health care in future.” | Supplementary materials S3 |
|  | **Domain 2: study design** | |  |  |
| 9. | Methodological orientation and Theory | What methodological orientation was stated to underpin the study? *e.g. grounded theory, discourse analysis, ethnography, phenomenology, content analysis* | Thematic analysis  Critical realist epistemology | Manuscript pg. 5 |
| 10. | Sampling | How were participants selected? *e.g. purposive, convenience, consecutive, snowball* | Purposive | Manuscript pg. 4 |
| 11. | Method of approach | How were participants approached? e*.g. face-to-face, telephone, mail, email* | Via mental health services | Manuscript pg. 4 |
| 12. | Sample size | How many participants were in the study? | 58 | Manuscript pg. 4 |
| 13. | Non-participation | How many people refused to participate or dropped out? Reasons? | 11 participants, across all sites  Reasons:   - Keen to "move on with life" - Keen to "focus on life" - Unwell - Despondent with healthcare - Not interested in using technology to monitor mental health - Scheduling challenges due to job hunting and other commitments | Supplementary materials S3 |
| 14. | Setting of data collection | Where was the data collected? e*.g. home, clinic, workplace* | In-person or via phone/video call, depending on participant preference and risk assessment | Manuscript pg. 4-5 |
| 15. | Presence of non-participants | Was anyone else present besides the participants and researchers? | Usually not. One participant’s partner was present. | Supplementary materials S3 |
| 16. | Description of sample | What are the important characteristics of the sample? *e.g. demographic data, date* | Described in results and Table 1 | Manuscript pg. 6 |
| 17. | Interview guide | Were questions, prompts, guides provided by the authors? Was it pilot tested? | Yes, topic guide in supplementary | Manuscript pg. 4-5  Supplementary materials S2, topic guide |
| 18. | Repeat interviews | Were repeat interviews carried out? If yes, how many? | No, one-off | Manuscript pg. 4 |
| 19. | Audio/visual recording | Did the research use audio or visual recording to collect the data? | Audio recorded | Manuscript pg. 4 |
| 20. | Field notes | Were field notes made during and/or after the interview or focus group? | Reflective logs | Manuscript pg. 5 |
| 21. | Duration | What was the duration of the interviews or focus group? | Range 20-78 minutes, median 43 minutes | Manuscript pg. 4 |
| 22. | Data saturation | Was data saturation discussed? | Not mentioned as not a relevant concept in this study, in which the sample size was determined *a priori*, ten participants per site to enable geographical heterogeneity. As Braun and Clake, 2019; https://doi.org/10.1080/2159676X.2019.1704846 argue, data saturation is not relevant to all types of thematic analysis, particularly reflexive thematic analysis, used in the current study. | N/A |
| 23. | Transcripts returned | Were transcripts returned to participants for comment and/or correction? | No | Not mentioned because not done |
|  | **Domain 3: analysis and findings** | |  |  |
| 24. | Number of data coders | How many data coders coded the data? | EE and 4 LEAP members | Manuscript pg. 5 |
| 25. | Description of the coding tree | Did authors provide a description of the coding tree? |  | Manuscript pg. 6 |
| 26. | Derivation of themes | Were themes identified in advance or derived from the data? | Derived from the data | Manuscript pg. 5 |
| 27. | Software | What software, if applicable, was used to manage the data? | Nvivo | Manuscript pg. 5 |
| 28. | Participant checking | Did participants provide feedback on the findings? | No | Not mentioned because not done |
| 29. | Quotations presented | Were participant quotations presented to illustrate the themes / findings? Was each quotation identified? e*.g. participant number* | Yes | Manuscript pg. 6-13; Supplementary material S5 |
| 30. | Data and findings consistent | Was there consistency between the data presented and the findings? | Yes | Manuscript pg. 6-13 |
| 31. | Clarity of major themes | Were major themes clearly presented in the findings? | Yes | Manuscript pg. 6-13 |
| 32. | Clarity of minor themes | Is there a description of diverse cases or discussion of minor themes? | Yes | Manuscript pg. 6-13 |

###

### S2. Topic guide

**Study title:** A qualitative study of service user and staff views on digital remote monitoring for unusual distressing experiences, psychosis

| **Participant ID number** |  |
| --- | --- |
| **Notes can be made on this topic guide. However, please do not include identifiable information and do dispose of any paper copies securely once the interview has finished.**  **When you finish the interview, you will need to complete a reflective log via Qualtrics**  **Please ensure that this log is completed after each participant and that actions based on the log are taken to the project management group as soon as possible.** | |
| **Prior to interview, research workers should:**  Send participant the consent form, in their preferred format and participant information sheet.  Check they have arranged a time to meet/speak with the participant and, if applicable, that a Zoom, or similar online meeting invite has been sent to the participant and that they have accepted.  The Research Assistant and participant should sit as close to the microphone as possible when using a dictaphone to ensure everything can be easily heard on the recording | |

**Introductions**

- My name is [researcher name]. I’m a researcher on the CONNECT study which is a study about using smartphones and wearable devices, like Fitbits, step trackers or smart watches to help people keep track of their mental health.
- Thank you for agreeing to meet with me today for the research interview. First, I just want to check in about how you are feeling today. Do you feel well enough to do the interview today?
- [If feels well enough, continue] Thank you for reading the participant information sheet and filling in the consent form. Before we get started, do you have questions about anything in the participant information sheet or the consent form? [Answer any questions].

Re-confirm informed consent is still valid and participant still wishes to take part. Key points to cover:

- Interview length: The interview today will last 30-60 minutes.
- Audio recording: We will audio record the interview.
- Anonymity: We will store the interview recording and all other study data securely using an anonymous number rather than your name. It’s best to avoid using full names in the interview, but all names will be removed when the interview is transcribed. We might use quotes from the interview when we publish the research but the quotes will not allow you to be identified.
- Limits of confidentiality: What you say in the interview will remain confidential unless something you say makes me concerned that you or someone else might come to some harm. In that case, I would have to pass the information on to your clinical team or another relevant person.
- Voluntary. It’s your choice to decide whether or not to take part. You can take a break or stop the interview completely at any point without giving a reason, and without penalty.

Are you still happy to take part in the interview?

*[If yes]* Ok, thanks, I’m going to start the recording now ***[Start audio recording]***

Note to research workers: **What follows is a guide.** The order and exact content of the questions will be determined by the participant and will be influenced by the ongoing analysis, so the order of the questions may vary as the interview develops. Prompt and ask for examples as time permits.

Explanations of key concepts and the main interview questions are in blue. Follow-up questions to prompt further discussion are included *in italics*.

| **Topic** | **Interview script/questions/prompts** | **Notes** |
| --- | --- | --- |
| **Overview** | |  |
| Overview of interview | In this interview, I’d like to explore what you think about using a smartphone or a wearable device to help manage your mental health. A wearable device is something like a smartwatch, a Fitbit or a step tracker. Here are some examples [Show Figure 1].  I’ll start out with some questions on what you think about this topic in general, and then go into some more specifics. Does that sound ok? | Figure 1: Example smartphones and wearable devices  Figures are provided at the end of the topic guide. Depending on the interview format, the figures can be:   - Printed on card to show to participants - Shared on the interviewer’s screen, if using an online meeting platform |
| Warm up Qs to set the scene and help the researcher know how to pitch later questions | Do you use a smartphone or a wearable device like a smartwatch, Fitbit or step tracker at the moment?  [If yes] What do you tend to use it/them for? *How often?, Daily? Less?*  What is your general view about smartphones and wearable devices like Fitbits, step-trackers or smartwatches?  *How do you find using them? Do you like them? Dislike them?* [If yes, follow up with an open question. E.g. What do you dislike about them? Can you tell me more?]  *Are there things that stop you from using devices like smartphones or wearables?*  *[If yes] Can you tell me a bit about those reasons?* | Note on personalising the interview:   - Briefly ask the participant what word they’d use to refer to a wearable – e.g. “wearable”, “wearable device”, “wearable gadget”, “wearable item”. Then use their preferred wording throughout the interview. - If the participant mentions a specific wearable, you can use that specific example throughout the interview, as applicable. If they use more than one device, make sure you ask questions about each device, as applicable |
| What are the barriers/facilitators to service users using digital tools for healthcare? | What do you think of the idea of using devices like smartphones or wearables for health purposes? That could be for physical health, fitness, or mental health…  Some people might use them for things like counting steps, helping with their sleep routine, setting medication reminders or ordering prescriptions. There are also special devices that people can wear to track things like their blood sugar or heart rhythm.  [If uses devices] Do you use your [name the device(s] for health purposes? Why/why not?  [If uses devices for health purposes] Do you find this helpful/unhelpful? *Can you tell me more?*  *Is there anything that gets in the way of you using technology like this for health purposes? What helps?* |  |
| **Symptom monitoring** | |  |
| Description of symptom monitoring | Smartphones can be used to keep track of your symptoms or how you’ve been feeling day-to-day by prompting you with questions.  I’ve got an example of an app that does this. It’s called the ClinTouch app. [Show Figure 2]  The app asks you about your mood and experiences, like whether you’ve been feeling low, and whether you’ve been bothered by voices. It can also ask you about where you are and the kinds of people that you’re with, family members, friends, etc, to check out whether certain situations, places or people change how you’re feeling. | Figure 2: Example symptom monitoring app question, ClinTouch app |
| What are service users’ concerns about active symptom monitoring and what do they perceive the benefits to be? | How would you feel about using an app to keep track of your mental health in this way?  *How would you feel about answering questions on an app about your mood or how you’ve been feeling? What would you find helpful/unhelpful?*  *How would you feel about answering questions about unusual distressing experiences like hearing voices or feeling very suspicious?*  *How would you feel about answering questions about where you are and who you’re with?, To see if any situations, people or places influence your symptoms.*  *Would anything worry you about using an app to keep track of your symptoms or feelings in this way? [If yes, what in particular?]* |  |
| How can we keep service users engaged with symptom monitoring? | How would you feel about tracking your mental health in this way long term? For example, for 12 months?  What would help you *keep on* tracking your mental health like this long term?  *Is there anything we could build into the app to help keep you engaged?* |  |
| Views on sharing information with clinical team | If you were to track your mental health using an app like this, your answers could be sent electronically to your mental health team: this could let them know if you need any extra support, for example, an extra visit or a doctor’s appointment.  How would you feel about this? Would you find this helpful/unhelpful? Why? *Can you tell me more about that?* |  |
| **Passive sensing** | |  |
| Description of passive sensing | We are going to move on to talk about passive sensing now. Smartphones and wearables, like Fitbits or smartwatches can gather lots of continuous information, without you having to *do* anything apart from wear the wearable or carry your phone around with you. This is called passive sensing. Here are some examples [show Figure 3 and give the participant some time to look at the examples of info tech can gather].  This information tells a story about the kind of things you’ve been doing – like whether you’ve been sleeping, exercising, travelling, or phoning people.  In turn, this can give clues about how you might be feeling. For example:   - Someone who is feeling very anxious or low might start socialising less. Their smartphone could gather information about their location and phone-use, that shows they are staying at home more and messaging people less, so it could spot that change.   What it can’t tell us, though, is why someone might be staying at home more than usual, or why someone’s sleep might be affected.  So, this process is different to using an app to record your experiences like we talked about before, because this information is collected automatically without you having to put any information into the phone or wearable device yourself.  Do you have any questions about how passive sensing works or anything I have just said? [clarify any uncertainty] | Figure 3: Types of information gathered by smartphones and wearable devices |
| What are service users’ concerns about passive sensing and what do they perceive the benefits to be? | What do you think about collecting information in this passive away? How would you feel about information like this being collected about you?  *Would you feel comfortable with this? [If yes/no] What in particular might you feel comfortable or uncomfortable about?*  *How might it help? What might the benefits be? Can you see any disadvantages?*  *Do you think your view might change over time – for example, might you feel differently about this on a day when your mental health was better or worse than today?* | Note: If they seem unsure or say they are not sure, clarify what the specific uncertainty is and whether they need more explanation about what passive sensing does and how it works |
|  | The information collected by the smartphone or wearable could be sent to someone’s mental health team too, so the team could be updated about changes in someone’s activity. The team could then offer the person extra support.  What do you think about this type of information being shared with people’s teams in this way?  *What would be helpful/unhelpful? Why?* |  |
| What can be done to make passive sensing technology more acceptable to users? | [If they have concerns…] Is there anything that could help you feel more comfortable about health-related information being gathered in this passive way?  [If they don’t have concerns…] Is there anything that you think we could do to help address other people’s concerns about this?  *Would you want to see a copy of the information the smartphone or wearable has gathered? [If yes] How would you want to receive this?, e.g. in the app, via email/post. How often would you want to see this?, E.g. just once, or on an ongoing basis?.*  Smartphones can gather information about where you are throughout the day.   - How would you feel about the app knowing your exact location? - How would you feel about the app knowing the general area that you were in, e.g. [insert relevant local area. E.g. Fallowfield]? - How would you feel about the app knowing how far you’ve travelled from home each day, without knowing where you were travelling exactly to or from? - *Which of these options would you feel most comfortable with?*   I am interested in what kinds of health-related information you would be comfortable with the smartphone or wearable gathering. Let’s look back at the examples we saw earlier [Show Figure 3 and give time to read the examples there]. Do any of these types of information cause you concern? Which ones? Why? |  |
| **Relapse** | |  |
| Description of relapse | People who have mental health problems like psychosis or schizophrenia may experience times when their symptoms worsen and become more problematic. This worsening is sometimes described as a ‘**relapse**’.  We want to help people to manage their mental health and minimise relapses. To do that we want to spot early when people are starting to relapse. That way, people can quickly get the help and support they need.  *[pause and check for understanding so far]*  We’ve talked about two different ways that technology might help people keep track of their mental health: 1 by allowing them to report their symptoms in an app; and 2 through passive sensing, where ongoing health-related information is gathered from the smartphone or wearable without the person having to do anything. In our CONNECT research study, we are looking at whether we can use these methods to spot early that someone might be getting unwell, before this is normally noticeable. |  |
| What are service users’ views on active/passive monitoring for *relapse prediction* specifically? | What do you think about using these methods to spot early signs that your mental health may be getting worse?  What might be helpful about this? What might be unhelpful? *Can you tell me more about that?*  *Would anything worry you about using technology to do this? [if yes] What would you be concerned about?* |  |
| **Machine learning** | |  |
| Description of machine learning | We are now going to move on to talk about a technique called machine learning. I’ll explain what I mean by that... So, using smartphones and wearables in the ways we’ve talked about collects a large amount of health-related information. For that information to be useful, we need a way of making sense of it, analysing it. One way of making sense of it is to use a technique called **machine learning.**  YouTube and Netflix use this technique. They gather lots of information about people’s viewing patterns and then make recommendations to viewers about what they might want to watch next.  In our CONNECT research study, we’ll use smartphones and wearables to collect a lot of health-related information from a lot of people with psychosis. Then we’ll see if we can use machine learning methods to pick up changes in people’s mental health.   - First, we’ll have a training phase where we teach a computer to recognise patterns in the information we’ve collected. For example, one pattern might be that people tend to leave the house less as their mental health is worsening. - In the training phase the computer gets lots of human guidance and supervision. - When the computer has finished its training, it can then spot patterns without human supervision.   Do you have any questions about this so or what I’ve said so far? |  |
| What are service users’ views on machine learning for *relapse prediction* specifically? | How do you feel about this process of a computer program learning to pick up changes in people’s mental health?  *Do you have any comments or concerns about this method? [If yes: how so? Can you tell me a bit more about that? What are your concerns?]*  *Is there anything that would worry you about a computer program picking up when you might be getting unwell? [If yes, what in particular?]*  *How would you see a computer program like this working alongside the usual care that you/other people receive from the mental health team? Would it feel different to the usual support on offer? Why/why not?* | Note: If they seem unsure or say they are not sure, clarify what the specific uncertainty is and whether they need more explanation about what machine learning does and how it works |
| What do people think about this information being shared with others? | If the computer picks up that someone’s mental health may be worsening, this information could automatically be sent a person’s care team. How would you feel about this? *What would be unhelpful/unhelpful about this?*  How would you want your care team respond to seeing an alert that your mental health is worsening? *What would be helpful/unhelpful? Why?*  How would you feel about an app electronically alerting a parent, carer, trusted other or supporter about those changes with your permission? *How would you want them to respond to this alert? What would be unhelpful/unhelpful? Why?*  How would you feel about an app alerting you directly? *What would be helpful/unhelpful about this? Why?*  *In general, how would you want to see this information?, e.g. in app alert, email, text* |  |
| What concerns do service users have around machine learning systems/ algorithms? | *What would you need to know to feel comfortable about services using this method as part of your healthcare?*  *What would we need to consider, from your point of view, to make this an acceptable way for services to support you? What could make things easier?*  *Is there anything we could do to address people’s concerns about using this method?* |  |
| What proportion of false negatives and false positives is acceptable for predicting relapse? | Just like people get things wrong sometimes, so can computer programs. The program won’t *always* get it right. Like with your care team, the computer might think your mental health is getting worse when actually it isn’t… and it might sometimes miss when you are becoming unwell.  What do you think about the idea that the program won’t *always* get things right?  *Do you have any concerns about this? Can you tell me more about that? Would this affect your willingness to use technology in the ways we’ve talked about?*  *Which would be worse for you – if the computer said your mental health was getting worse when you were actually fine… or if the computer didn’t pick up that you were actually getting unwell? Why is that?*  *How should we tell people that the computer got it wrong? How can we manage this?*  *(What do you think about the idea that your doctor gets things wrong?* |  |
|  | |  |
|  | In future, a system that uses smartphones or wearable devices to help people keep track of their mental health might eventually become part of the standard care offered by mental health teams, so that everyone under the care of a mental health team would use a smartphone/wearable system in the ways we have talked about.  What do you think about this?  Do you think should people be able to opt out of using a system like this? Why/Why not? | [Note: this is intentionally controversial to get participants thinking about what they would or would not be happy with] |
| **CONNECT** | |  |
| Consent procedures  What concerns do service users’ have around data sharing and trust online? | What would you want to know before you agreed to track your mental health using a smartphone/wearable?, *e.g. storage location/duration, who has access*  *If someone was going to start using a computer program like this as part of their mental health care… what do we need to tell them?*  *How much detail would you want to know about how the information was being stored and used?*  How often would you want to be reminded that information was being gathered from your smartphone or wearable in order to check that you still agree to this?, *e.g., Monthly? Every 3 months? Every 6 months?* |  |
| Views on ethical/ governance issues associated with data tracking/storage in a digital health context | What could we do to help you trust this kind of system?  What do you think we could do to help you feel confident that your information is being stored safely and securely?  *Who should have access the smartphone or wearable information?* |  |
| Is the proposed digital health data management plan for the CONNECT study appropriate and acceptable? | In our CONNECT research study we will collect information from smartphones and wearables to use in the ways we’ve discussed today. We will store that information for at least 10 years in a safe, password protected folder on a university or NHS computer system.  How does this sound to you?  *Does this sound safe? Acceptable? Secure? Is there anything else we should be considering? Is there another way we could do this? [If yes, How?]*  Would you be happy for other researchers around the world to have access to anonymous information to answer important scientific questions?  [If no] What are your concerns about it? |  |

Interview closedown

- Is there anything else that you would like to tell me that we haven’t discussed?
- How have you found this interview today? How are you feeling now that we’ve done the interview?
- How do you think this interview could be improved for future participants?
- Ok, I'll now switch off the audio recorder ***[Turn off recording]***

Thank you for taking part in the interview. Just before we finish I’ll go through some very quick demographics questions with you if that’s ok – just about your age, employment status etc.

***[Go through demographics Qs and input directly into Qualtrics]***

Finishing

- Thank participant for taking part and let them know that their contribution to this research is extremely useful and important.
- Explain what will happen now and how this information will be used
- Offer to provide summary of the findings when available
- Offer a phone call: Sometimes people take part in an interview and afterwards they have more questions to ask, or they have been worrying about something they said. If you like, I can call you tomorrow or the next day just to check if any of this is happening for you – would you like me to do that?

***[Complete reflective log via Qualtrics]***


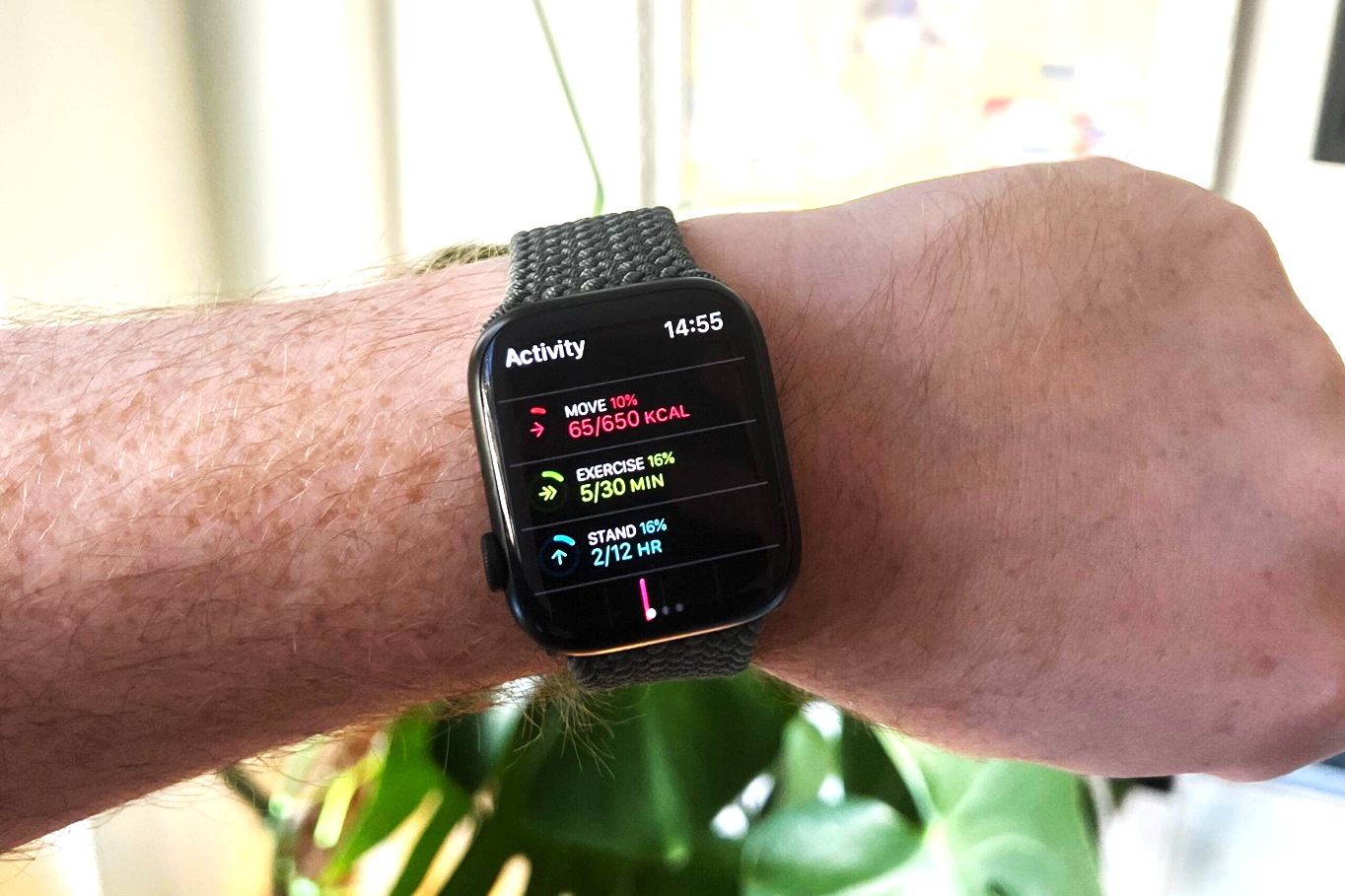

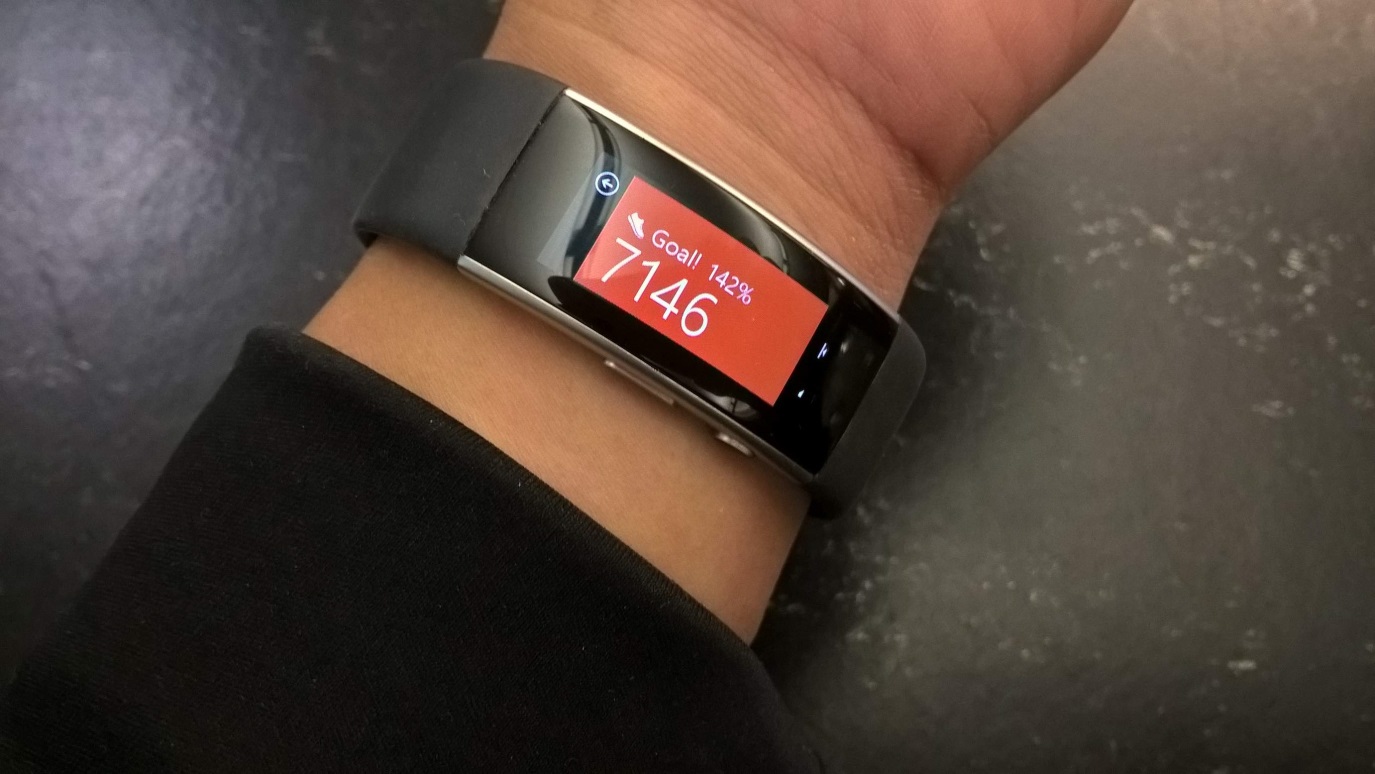
***Figure 1: Example smartphones and wearable devices***


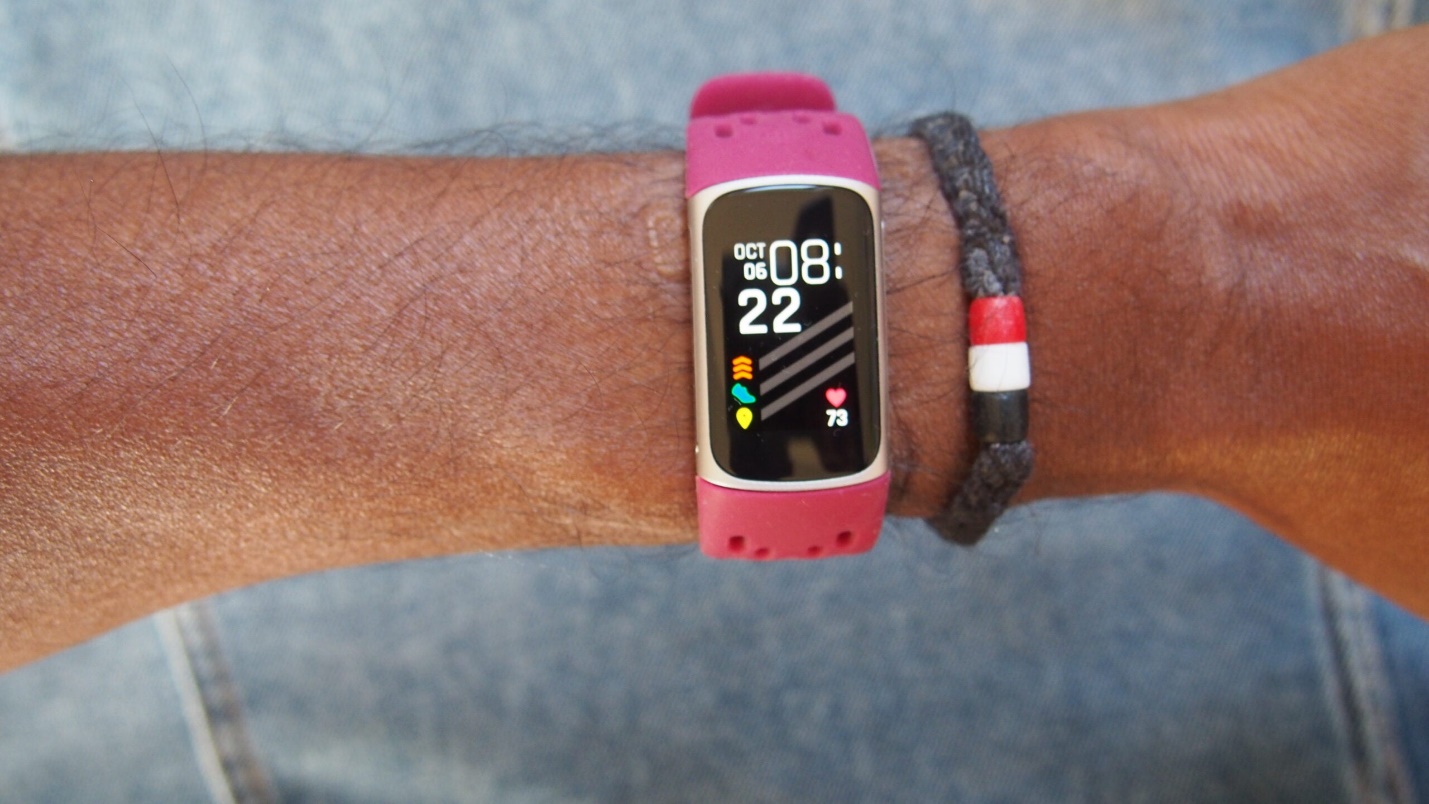

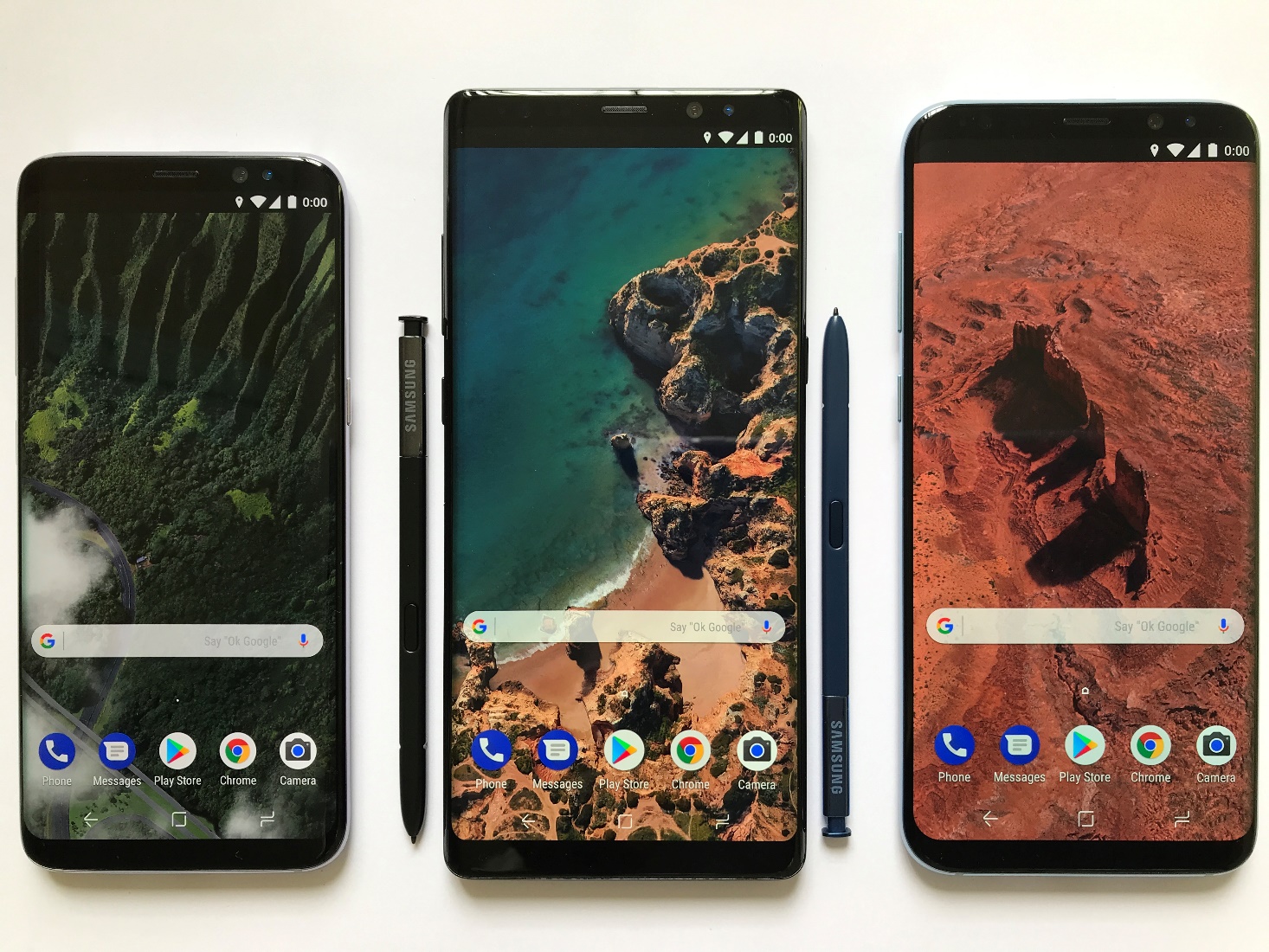

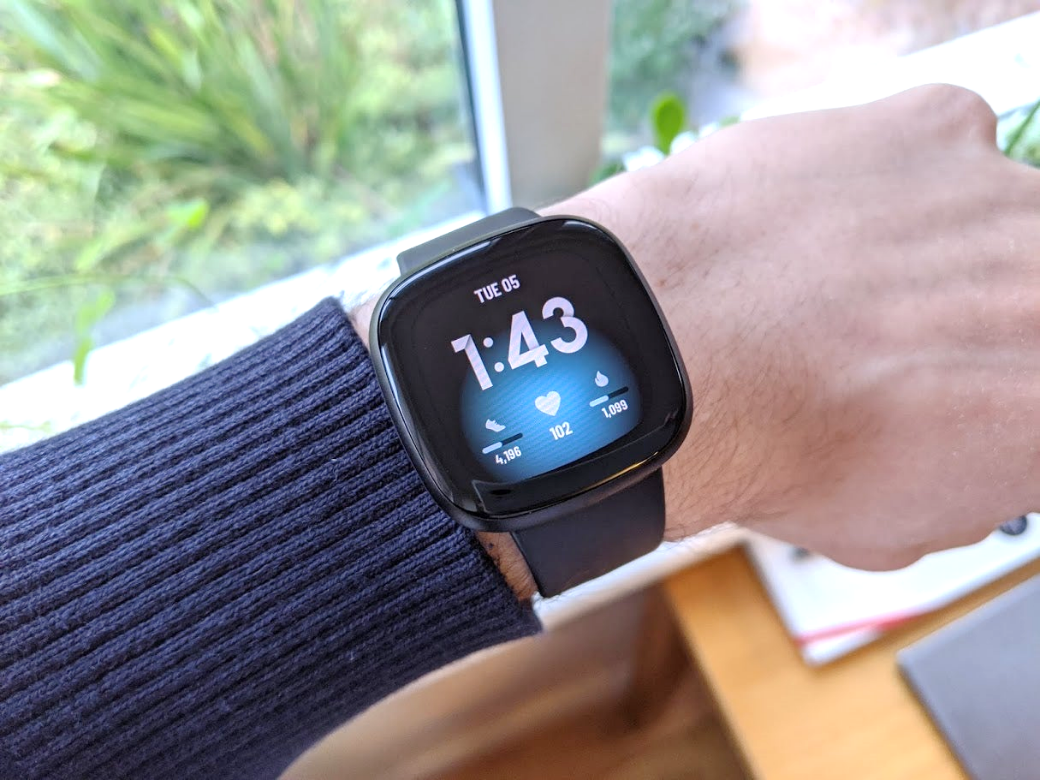

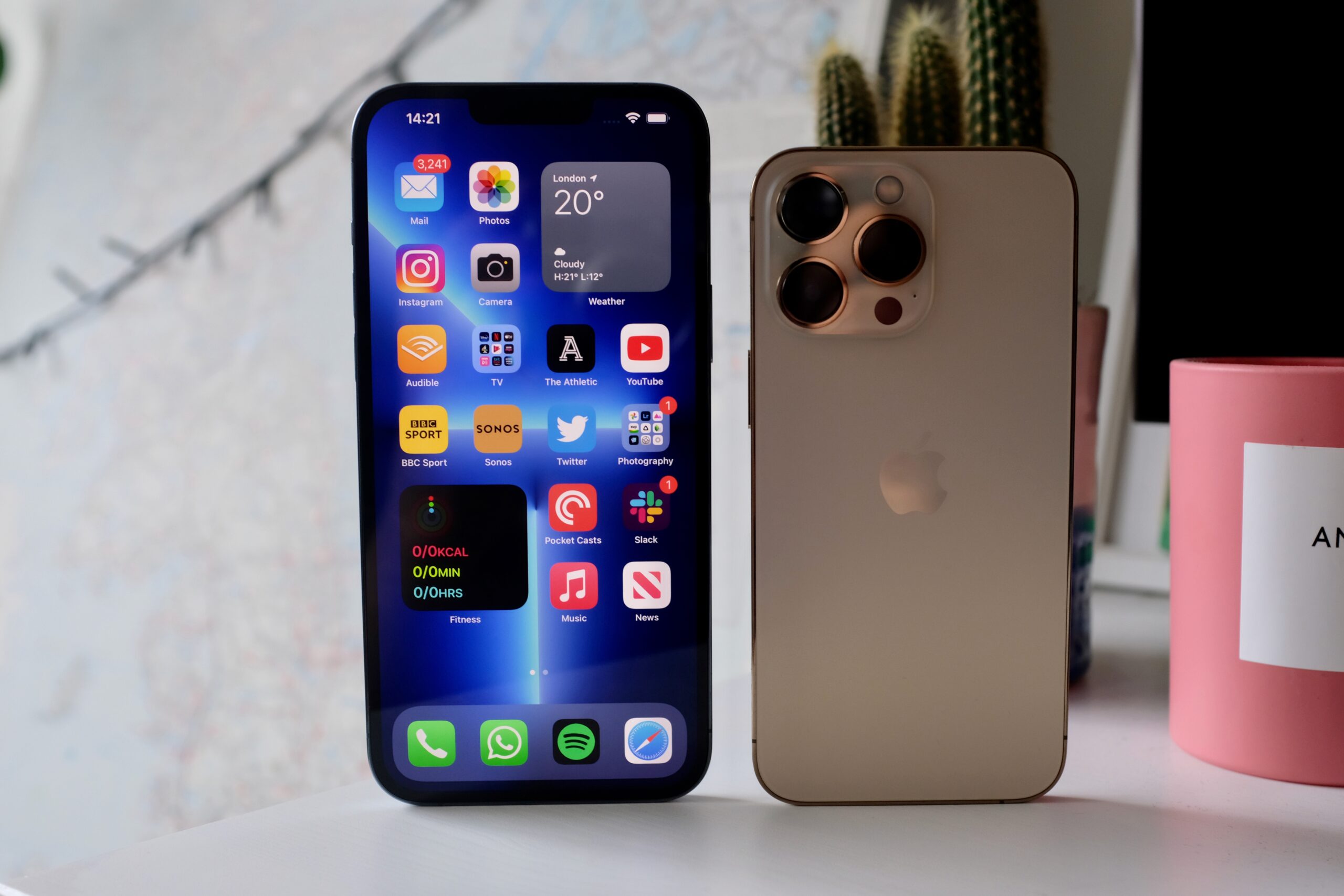


Smart watches Step/fitness trackers

**Smartphones Wearables**

***Figure 2: Example symptom monitoring app questions, ClinTouch app***


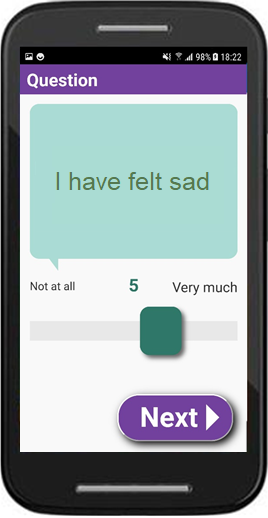


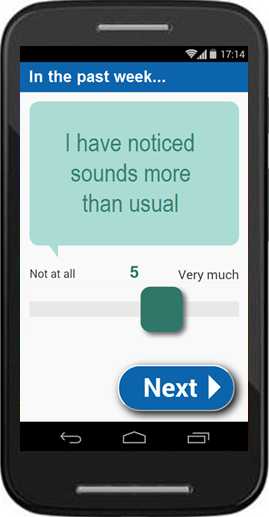

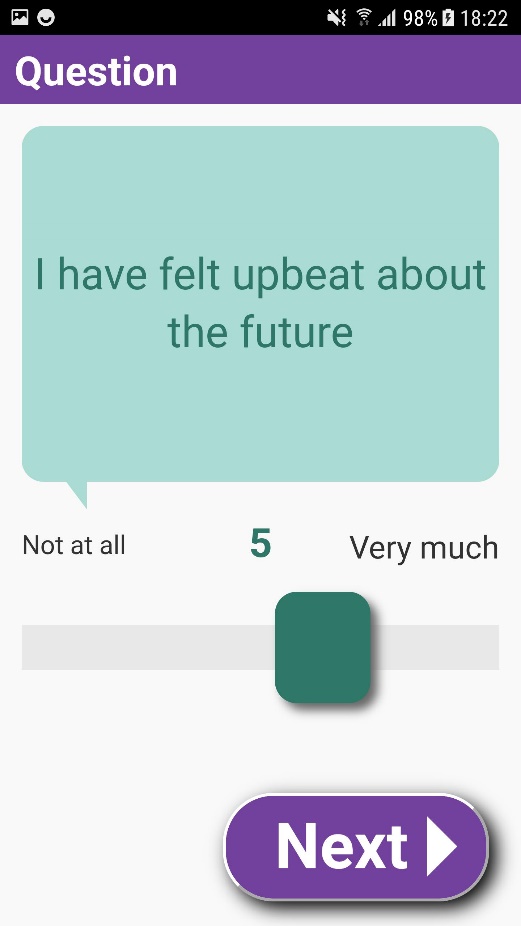

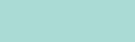


I have heard voices

***Figure 3: Types of information that can be gathered by smartphones and wearable devices***


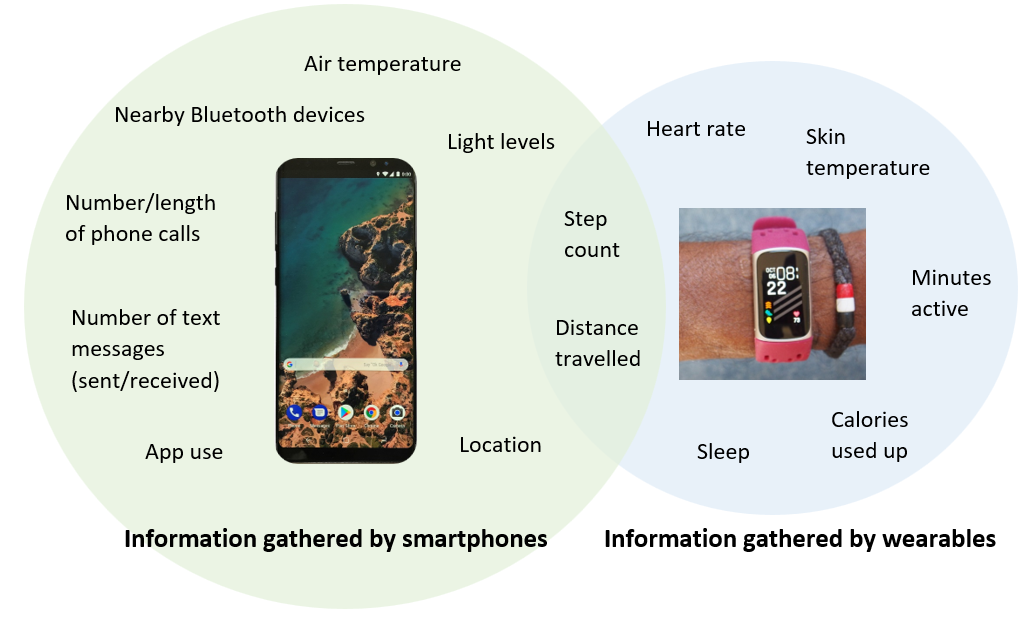


### S3. Supplementary methods

#### Epistemological approach

Consistent with a non-positivist epistemological stance, no attempts were made to establish inter-rater reliability. This approach acknowledges and values researcher and lived experience subjectivity as an integral component of the interpretive process, recognising potential bias rather than seeking to control or eliminate it.^29,32^ Data analysis was underpinned by a critical realist epistemological approach, which is particularly well-suited for exploring complex social phenomena such as the implementation of DRM in mental health services. Critical realism assumes that objects, including social objects, exist but that our attempts to describe them are fallible.^33^ This approach acknowledges researcher and participant subjectivity but allows tentative transferability of study findings by aiming to explain and understand events beyond the study sample.^34^ This epistemological stance is especially valuable in the current study, as it facilitates a detailed exploration of participants’ experiences, acknowledging both their subjective interpretations and the broader structural and contextual influences on DRM adoption. For example, participants’ diverse prior experiences of mental health services (e.g., supportive, paternalistic, or coercive) are likely to affect their views on using DRM in this context, as are their experiences of wider issues such as societal stigma.

#### Reflexivity

All researchers were part of the CONNECT study research team which included academics, researchers, clinicians and people with lived experience of psychosis. Researchers who completed the interviews were primarily employed as local site researchers on the study. Interviewers, n=9; CR, HB, KO’H, LM, NC, RT, SC, SF, UZ were all women, and all had relevant doctorate/master’s degrees. Interviewers received study-specific training by SB, HB, and EE, who are experienced in qualitative research methodology. Training covered general background to qualitative research, interviewing skills and role-play interviews with feedback. HB, a female Clinical Psychologist and PhD researcher investigating the implementation of digital technologies in psychosis care, provided supervision to interviewers, which included listening to interview recordings and providing feedback.

Most participants did not have pre-existing relationships with interviewers, but a small minority knew their interviewers in a professional capacity. One participant’s partner was present during the interview to support the participant. Researchers conducting the interviews or analysis had some background knowledge of the topic and likely brought assumptions and biases, e.g., feeling invested in the idea of a DRM system being used in future mental health care. All participants were aware that interviewers were researchers involved in the CONNECT study and thus may have assumed an inherent interest and favourable bias towards using digital technology in mental health care.

#### Eligible individuals declining to participate

Across all sites, only 11 of the 69 eligible individuals who were invited to be interviewed declined to participate. Cited reasons included being keen to "move on with life", being currently unwell, feeling despondent with healthcare, not being interested in using technology to monitor mental health, and scheduling challenges due to job hunting and other commitments.

### S4. Health apps installed on participant devices

Word cloud of health apps that participants reported were currently installed on their digital device. Larger text indicates more frequently reported apps.


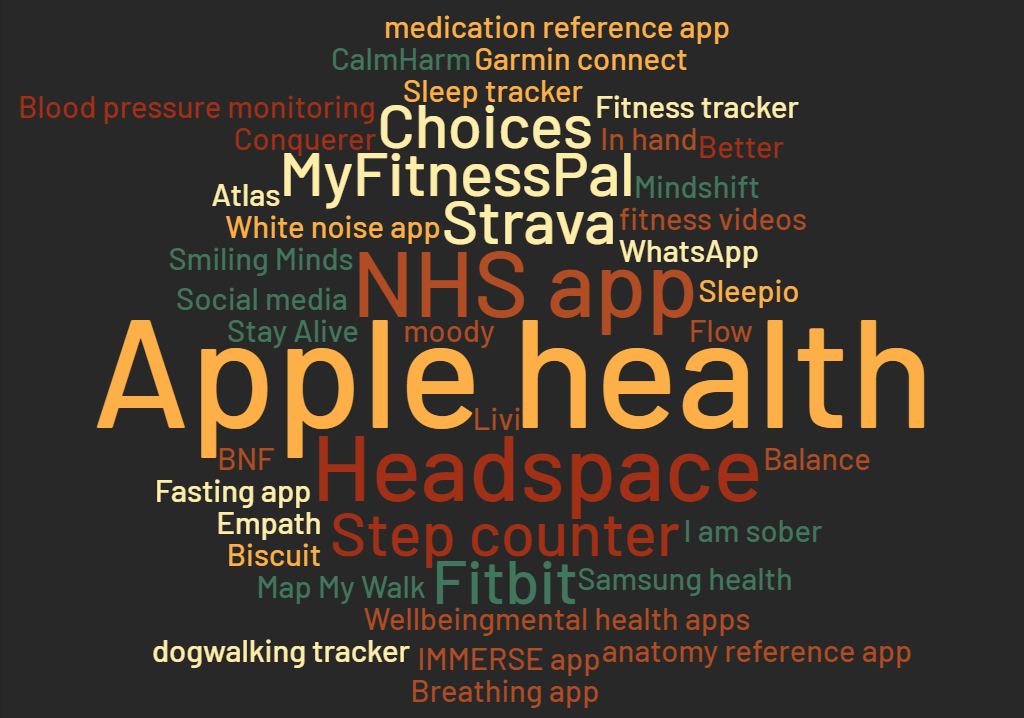


### S5. Supporting quotes per theme

| **Theme or sub-theme** | **Participant quotes** |
| --- | --- |
| **1. Mixed reactions to privacy implications** |  |
| 1.1. Visceral reactions to potential privacy violation | - “People are going to feel like it’s…they’re asking for too much information”, K006 - “I just think it’s intrusive, you know, you can lead a natural life, you don’t want to be hooked onto every device that’s coming your way”, K002 - “It kind of makes me afraid because I think now, I think about it like there's so much collected. Yeah, and these smartphones and there's so many things that come into it you know, sleep, how many hours you've been sleeping or how many steps you've taken. Yeah, you know, your locations and stuff that you can pick up on the background and show you on the map where you've been, I think, I think, I think for memory or for convenience it's good, but I think again, relating to psychosis, that could be scary for someone and it might even push them away a bit more because they know that their phone is capable of doing that”, C004 - In response to a description of the type of data that smartphones and wearables can gather: “Jesus Christ”, K008 - “The sleep, plus the minutes active, plus all the rest of them, just, that’s the Matrix. Oh my head.”; “it’s already happening now. Google is doing that. That’s Matrix stuff right there”, K008 - “It’s a bit offensive, like because I don’t want to know like…others to know what’s… who I’ve phoning and where I go”, G009 - “What you are asking me to do is basically wear a wire”, K008 - “It’s like you’re being tagged by the police, like you are on parole”, E001 - “It’s a lot to give away, because now, you don't have a private life anymore… it's just taking everything. If you are willing to pay me big, I would do it”, M006 - “There’s no way you could motivate me to do this”, K008 - “Take out all the invasions of privacy bits”, K008 |
| 1.2. Minimal concerns for some | Reasonable cause   - “As long as it's going to be for a good reason, obviously”, C001 - “I think if it's used for the right purposes then there shouldn't be an issue”, C005   Not too personal   - “It’s nothing that is going to harm a person, so I would agree it would be ok”, G010 - “I can’t see anything from the data and that would be a risk to my personal safety or identity”, K003 - “I think everybody always has concerns, who’s gonna have access to data and stuff like that but it’s not too personal, it’s not like your text messages are getting read or whatever”, E004   Purpose of devices is to collect data; used to it   - “My generation from I suppose mid 90s onwards have grown up with… being fully aware that the device is in your pocket and what you wear are collecting lots of data on you”, C005 - “I, kind of, track the stuff myself, like hours spent on my phone and what I’ve spent it on and what not”, M004 - No concerns with a device gathering data because “that’s what it does”, K010 - “I…track the stuff myself”, M004 - “I usually have my phone location on anyway. So you get alerted when you visit websites as well, or Google. So I think that’s fine”, C001 - “I think all the apps know where you are so why I would…Why that app would concern people?”, G005 - “My phone knows that so… Might as well be in the app”, K010   I have nothing to hide   - “Ach I’m pretty, squeaky clean, you know that, I don’t think there’s any… anything for me to to be worried about that”, G002 - “I’ve got nothing to hide so, you know, but there’s other people who’ve got something to hide who’ll say that’s too much”, E003 - “That wouldn’t bother me either to be honest, because… Yeah, don’t go anywhere wrong, I go to work, I stay in my flat, I don’t – yeah”, S001 - “I’m not really bothered about that, knowing where I am. I’m never anywhere exciting”, E004 - “I’m always in the same places”, E007 |
| 1.3. Uneasy feelings about location tracking | Uneasy feelings   - “If it were exact location I'd I would be a bit wary of that really... it just sounds like really like big brother to me… That might sound old fashioned but that's just yeah, I'm not convinced that's a good idea… Because it's open to abuse, I'm not saying that that would happen but It's just… it gives me a bad feeling about that kind of thing”, S004 - “People looking at exactly where you were, it’s like it’s a bit personal, that. So yeah, I’d feel a bit uncomfortable with that”, M010 - “That’s a bit worrying… Like, knowing my exact, who knows?... You don’t know who knows”, K007 - “I’m just a private person... the thought of my movements being tracked just doesn’t feel comfortable”   Right to privacy   - “I've got choice about where I go, it's, I shouldn't have to be monitored”, S004 - “I just feel like humans, we don’t need everything tracked, and it just feels a bit like intrusive”, K004 - “Like you’re being watched”, K004 - “I want to be able to go places and nobody knows where I am”, E001 - “I think what I’m doing during the course of the day is what I want to do and it’s no one else’s business to be shared with a clinical team that might analyse it negatively”, K002   Nothing to hide but still want privacy   - “Yeah, I would feel that’s maybe a bit too personal. Not that I’d get up to like anything mischievous or anything, but yeah”, M010 - “it's not like I have anything to hide, yeah. But sometimes, one needs to keep life private, just some things private”, M006 - “Well it's if if it knows where you are and what you're doing all the time, it's a bit, bit big brothery...I mean not that I'm going anywhere that I shouldn’t be. Ahaha…I just don't like the idea of somebody being able to say oh she's in Tescos, y'know”, S007   Interplay between location tracking and existing paranoia or anxiety   - “Relating to psychosis, that could be scary for someone and it might even push them away a bit more because they know that their phone is capable of doing that.”, C004 - Re why passive monitoring might worry someone: “Might be privacy… knowing me in my early years, I’d be like oh my god they’re spying on me, they know my location, they know my text message, you know what I mean. With psychosis you go wooop off the wall, if you know what I mean. Paranoia. So them ones would have to be explained and as long as you’ve got consent for them, and you explain that you’re not listening into the phone calls or you’re not reading the text messages, they might be happy in regard to that, if you know what I mean”, C007 - “I don’t fancy being tracked. I could get a bit paranoid and start leaving my phone at home, that kind of thing”, M001 - “I don’t just feel in control of my situation and life anymore, if an app is to know. It could give room for mental health problems”, M002 - “Myself, personally, yeah, I am happy with that, especially if I’ve sort of said yes to it, I do know people however who can get quite paranoid about that sort of tracking”, S008 - “I’m funny with location I always turn mine off” because “I think someone’s following me sometimes”, S002 |
| **2. Autonomy, freedom and control** |  |
| 2.1. Fear of judgement or embarrassment | Sleep   - “I still feel like, in a way, either way, I wouldn’t want to take part… [it’s] too much [information]… I don’t have a set sleep pattern”, K008 - Not comfortable sharing sleep information because “My pattern’s all over the place”, M009 - Participant joked that they might get “told off for sleeping too much”, C003   Substance use   - “Could be doing something with drinks like you’re always down Wetherspoon’s on a Saturday having a drink and then you’re feeling miserable the next day you know”, C007 - “If I was still taking drugs like I used to in the past I would have said no to [passive sensing] aye but I’m not on drugs anymore so I wouldn’t mind”, E003 - “When I was in my early 20’s I used to… go to people’s houses and take drugs and stuff like that, so I’m not sure if I’d want to be recording all that for the psychiatrist because it you know, they’d frown upon that”, E009 - “If they know you get drugs in [place name]…and they see you are going down to [place name] and you’ve told them you’re not taking drugs then that’s one example of a problem”, E009 - “I don’t have much to hide at the moment but like I say when I was younger you know if I was visiting drug dealers and things like that I probably wouldn’t want specific information to be you know showing ‘aw he’s going up there”, E009   Relationships   - “Numbers of text messages sent and received, maybe they’re like oh I don’t want you to know that I’ve been texting [fictional name] I’m in a relationship”, C007 - “Someone might not be in a good relationship and they’re spending time there...And where could it go anyway …You’re not going to offer domestic violence help…so it could get very tricky”, K002 - “You might be having sex with someone you know, you wouldn’t want them to be in on that”, E009 |
| 2.2. Perceived pressure to change behaviour to gain clinician approval | - “To have all your daily activities monitored and relayed back to someone who’s treating you seems to me like a curtailment of my personal freedom”, E001 - “Once you know that all your life’s being relayed back then it could influence how you live...I think that’s a problem”, E001 - “Imagine it stopped people from doing things they wanted to do, if it was to go out for a night or something or…the psychiatrist knew they hadn’t been sleeping as much as they should of, but maybe they felt they…didn’t need as much sleep. To me it just seems like you’re making more of a subject of the patient and less of a person”, E001 - “Imagine somebody’s daily activities are being monitored and then they feel like ‘oh well I need to make a go of it to show the psychiatrist what I want the psychiatrist to think, so I’m gonna get up and I’m gonna increase my steps and I’m gonna meet more people and I’m gonna sleep more and I’m gonna live my life according to the data’. I think that will be a problem”, E001 - In response to the idea that the clinical team or app might prompt them to go out for a walk, if passive sensing information indicated sedentariness: “Yes that’s good, yes…But I’d be annoyed as well, because I like to play games. So, when I’m always…I’m always at home, really. Well, I try to be at home”, K006 - “If its constantly going out to…my keyworker or whatever then I don’t think I’d like it cos I’d feel like I’d have to go out some days, even if I didn’t want to, just so it shows…I had been out”, G008 |
| 2.3. Wearables promote behaviour change | Pressure from device itself; prefer not to receive data   - “I guess it is why I maybe only wear my Fitbit if I’m doing exercise, cos I don’t want to have some sort of like micromanaging thing…I don’t think I would want to be like notified about like all the passive information that is being gathered cos most people are quite happy how they’re going, they don’t want any external information to influence how erm how they are doing their day-to-day tasks”, G008 - “If I wanted it I could request [information from the wearable] somewhere I’d imagine but I’m happy with literal ignorance, happy not knowing”, G008   Danger of becoming obsessed with wearable data   - “It can get a bit dangerous if you track like everything you do”, G008 - “Fitbits I only use it for fitness, erm but I guess for health I could like erm like sleeping or I don’t use it to track like what I eat as I think there’s some element of it can get a bit dangerous if you track like everything you do…” - I’ve used like different apps to like erm help me sleep and eh some like mental health apps as well, I think you could qualify them under, erm, but I try not to like micromanage it though”, G008 - “I don't really use it that much for that because I kind of don't like checking. I know people who check their smartwatches for, you know, their heartrate and all of that and they get their…I think some of them even give you like an ECG or something on your phone. Sometimes I think it kind of like contributes to a bit of health anxiety, I don't know. I think it can be helpful definitely if you've got an actual physical health problem and you need to be checking say your heartrate or whatever it is. But I think sometimes, you know, it can become a bit like too much and you just, yeah, end up sort of fixating on it a bit some people. But…so that's why I don't check it as often”, C002 - “I think if I used it more, you just get a bit obsessed don't you just like with your numbers and stuff. Or maybe this is just me but, you know, check your numbers all the time and I don't know, yeah, I definitely think sometimes it would contribute to health anxiety, yeah”, C002 - “It’d make me neurotic, I’d become a hypochondriac, I think”; “Because if I knew that I had high blood pressure or whatever they look at, then I’d be at the doctors all the time”, K002 - “It was triggering me to be honest with you. I got like obsessed with like the gym and calories and stuff when it just- it made me just a bit poorly so I had to stop using it.”, M012   Valuing passive/wearable data to support behaviour change   - “So, like when they notice it, it kind of like motives you like oh I’ve not been out in a few days, I should probably go out”, M012 - “Because I was tracking it, it made me more motivated to increase my steps and get out the house more and just go for walks. So I think it helped me”, C001 - “You set a distance… Then at the end of it you get a medal to say you have done it”, G001 - “I’ll be walking along, or running along, and suddenly my Fitbit would…start vibrating, because I’d done so many steps, and that was quite nice… a little burst of, oh, well done, from my Fitbit”, K001 - “if you had your Fitbit on and then someone went for a run and then they’re thinking oh how far have I ran, they can tell you and then next time you’ll think alright I’m going to try and beat that, like you know, they’d have little goal”, M012   Mixed experience of wearables, sometimes oppressive, sometimes supportive depending on mental health   - “In one mindset… it’s a good thing, you know, there’s loads of benefits to it…but then at the same time… when you’re not in a well mindset…It can kinda do the opposite”, M012 |
| **3. Suggested solutions to address privacy concerns** | |
| 3.1. Choosing data types to share with the DRM system and clinicians | Choose to opt in or out of certain data types   - “Having options maybe that we can turn this bit off if you’re not comfortable about say your location…we can compromise on that”, M011 - “In the settings of the app…it could have…the type of information you allowed app to… gather…like location…if you want you could disable that”, G005 - “I think it would be good to have the option [to decline certain data types], but myself I’d be happy to just do it anyway”, S008 - “I don’t think I would [disable location sharing], because I would like to track...where I am and stuff…but, just to have the power to be able to…”, G005 - “If there were filters in place, then I wouldn’t feel worried about it because I’d feel in control of what’s being sent”, M008   Separate into health data and personal data   - “I think having different permission levels on the app, like being able to filter the information that’s sent or at least the types of information that are collected…that would help or separating it into physiological symptoms and then, sort of, day-to-day personal things like number of calls, texts, messages and location, I think they should be distinguishable and you should be able to choose which side of the app you want to use”, M008   Ok for me to share in person but not digitally   - “It’s a bit offensive, like because I don’t want…others to know…who I’ve phoning and where I go. It’s something like I would like to share like in a face-to-face meeting but not like only through the electronic devices”, G009 - “it’s not right for them to get hold of your information all the time, like without you knowing, or, it’s okay for you to share the information with them, that is not right for them to like go on your phone and stealing information, and using your data against you, rather than helping you”, E005 - “The problem is, is annoying for someone else to know everything about you, what you doing, and what you carrying, who you texting, it’s like, it taking your independence away, like every, like you just, its making me a bit I don’t know, not feel right, for someone else to get a hold of your data. Doesn’t matter, it’s okay for me to show, but it’s not right for someone to”, E005   The clinical team could request information via the app   - “Probably they can just ask like through the app instead of just keep tracking? Through the… like through the smartphone”. - “If I can choose, like sometimes when I feel really unwell probably I would click yes”, G009 |
| 3.2. Temporarily stopping data collection or sharing | - “If I can turn it on and turn it off, yes, I’m fine. But I don’t really like to let everyone know where I am”, K006 - “Overall, it is a good idea but I’m stuck with all the privacy thing that maybe there’s a button you could press on the phone that ceases all activities ‘til you press it back on again. Not just the on/off switch, but a button that you could press that gives you privacy”, M003 - “I think it should give the person a choice to turn it on or turn it off because it can be a bit intrusive, you know. You can have a time where you don’t want anybody to know what’s happening in your life and you can be able to handle it tomorrow”, M005   Participant C002’s suggestion to choose each day whether to wear the wearable   - “In the morning…maybe have an option for the day if they want that on for them to see. Or if they themselves want that to be sent. And then if they're feeling like they don’t want to…then they can just click no…and then it doesn't do it for that day. But [it] also lets the clinician know that they're doing it because they want to, not because they're avoiding it”, C002 - “It's just giving a bit more independence…towards their treatment. Not just to have this on them and then they do it every day and they don't have a say whether it gets to the clinician or not”, C002 - “Otherwise, it kind of feels it's taken out of your hands and then you don't have the autonomy to do it yourself and almost puts it onto the clinician to always be tracking your emotions…it might just cause a bit of reliance I think on healthcare providers”, C002 |
| 3.3. Sharing less precise location data, deductive sub-theme | Prefer general area   - “If it’s constantly going out to my keyworker… that kind of general area, that wider circumference is probably better than having a pinpoint location”, G008 - “Yeah. Generalising the whole thing would be better”, M005 - Prefer general area to exact location because “I’m funny with location I always turn mine off”, S002 - “I think a general area would be a better one because then it feels less intrusive”, S005 - General area: “That would be okay”, S007   Prefer distance travelled   - “Actual distance is fine. I know a Fitbit gives you an estimate of that, it is not based on GPS, it is just based on steps…I suppose I am just concerned about battery running out, practicalities”, G001 - “I’d feel quite…content with [distance from home]. I wouldn’t feel too disturbed by it because it, if they’re not pinpointing specific precise locations, it wouldn’t really be able to have as much… in-depth knowledge as if it was able to monitor the exact whereabouts”, K003 - “From my own experiences I stay in a lot... When I'm going into [an episode] … and it would be quite interesting to know how far I've left my house”, S006   Worries that exact location could be inferred from distance travelled   - “I’m a conspiracy nut…any distance you put into walking speed…you can still work out roughly where someone’s going”, K008 |
| 3.4. Managing ongoing consent for passive sensing | Importance of informed consent   - “People were allowed to be given an informed decision, you know, with as much as the information that could be provided to them as possible. Then from there I suppose it's up to the individual, C005 - “As long as they give consent to do it then it’s fine innit”, C007 - “I’d like to know everything about it. I’d like someone to sit down, like we are now, and talk until… it’s been explained the best way possible for me to understand. And then I’d make a decision. But I’d like it to be objectively not all positive to…the phones and the apps. We’ll do all this with silver bells when it doesn’t tell you that it’s tracking your location and what you’re doing”, M003 |
| **4. Weighing benefits against privacy concerns** | |
| 4.1. Cost-benefit analysis, trade-off | Mental health benefits outweigh privacy concerns for some:   - “It’s obviously taking a bit of your privacy away right? If that’s what you’ve gotta do to keep yourself mentally stable then that’s fair enough innit”, C006 - “It’s good for mental health but there might be invading in privacy, that could be the only concern… But I’m okay, I’m okay in my situation”, C007 - “People might think it is an intrusion of their privacy or some people might feel like that but… I would feel like I’m being watched over which would be good for me actually”, M014   In contrast, benefits did not outweigh privacy costs for everyone:   - “I don't mind a bit of intrusion if it's gonna help you, I think that's taking it too far”, S004 - “I think in theory, it's a good…it definitely would be really helpful for clinicians to see…if they're staying at home doing all this. But I think the only thing for me would just be like privacy”, C002   Personal decision   - “Each person has to weigh it up for themselves and figure out what’s more important for them”, K004   Some people take time to recognise the benefits:   - “Yeah, eh the more I’m thinking about it the more I think it’s a good idea, even though I didn’t say that at first so…Maybe I’ve spoken too hastily”, E001 - “Participant's opinions seem to develop as the interview progresses. Initially she is hesitant about providing lots of passive data, "it's a lot to give away" and would only do so if very well financially reimbursed. Later, though, she says "it's fine because you have a lot to gain", which contrasts with her opinion that it's a lot of data to give away”, analysis diary notes on M006 interview |
| 4.2. “I don’t see the point” of passive sensing | - “I just think it’s intrusive, you know, you can lead a natural life, you don’t want to be hooked onto every device … things like calories used up and sleep, and heart rate, and skin temperature, minutes active, you know, it’s making you look at too much”, K002 - “I don’t see the point in having it dictate everything and every move you’re making and every breath you’re taking”, K002 - “I just think it’s too much information, where does the information go and what will the result be, and why are you collecting it every day anyway”, K002 - Re location tracking: “I don't need an app for that. I don't need an app to know. It's a redundant, there's no point for me”, K005 - “The premise here is that it would help me to observe my activities which would provide an explanation to my emotions. However, I feel like the monitoring of the activities could be done by me. I feel like the explanation of the activities could be done by me”, E001 - “Umm I think it's just me as an individual because I don’t feel as though I've got too much going on in my life that, that, that's problematic so I don't feel the need for it so”, S004 |
| 4.3. Specific benefits of passive sensing | Ease   - “It just makes things a lot easier because you don’t have to plug in specific numbers into a phone, it just sort of does it for you”, M010 - “The passive way is better… Because then, ‘cause I remember when I was at my worst, I don’t know if I would have been able to engage with my phone”, K007 - “I think the fact that it’s not a manual input makes it a lot easier especially for people that are experiencing low mental health whether it’s like mood or anxiety or psychosis. I think, like I said before, if you’re not in a state where you feel up to proactively doing something, it doesn’t matter because the information is just being gathered automatically”, M008 - “I think for memory or for convenience it's good, but…relating to psychosis, that could be scary for someone and it might even push them away a bit more because they know that their phone is capable of doing that.”, C004 - “I think it’s very convenient”, K003 - “I didn't have to do much…Y'know and it was just a case of wearing it… in the background…so that didn’t worry me at all”, S007 - “I think it’s a good idea, it’s definitely like, it’s essentially building evidence in the background like you don’t have to try and track it yourself, which is a useful thing”, S008   Flagging possible relapses   - “Moving around possibly too much… getting up in the middle of the night or, or doing going out and doing getting into kind of a bit of a mess going, going into places where you shouldn’t be going… or the other extreme just not going out at all”, S004 - “I'm not convinced about this GPS thing knowing exactly where someone is at all times it's, it's too intrusive really… but… if you're stuck in the same place…if you're just basically lying in your bed all the time… and it registers that you're not moving, you're not sort of going out and engaging with the outside world then that would be helpful because I-I have been through that myself”, S004 - “If I’m not open to tell someone where I am, at least they can find out from another way”. M004 - “When we suffer with mental health we close ourselves in. And we don’t wanna inform other people ‘cus we don’t want them to worry. But then at the same time, we’re isolating ourselves and we’re not interacting with anyone, so yeah that is good with the location”, C007 - But decontextualised data may cause false positives:   - “I think it’s a good idea, but it would do nowt with me ‘cause I only go out for about ten minutes a day”, M009   - “Really depends on… how much you have to do and where. If you’ve got any other outdoor resources like… mental health or workplace or voluntary or whatever”, E006   - “say if someone doesn’t leave the house and it’s not to do with their mental health, would that skew their result, would that impact it?”, C004   - “I think the issue would be then it's it's knowing what to do with that information, so a lot of that information will probably come in. Quantitative form that, you know, it's a lot of numbers figures that need to be put into categories and yeah, whereas I think that maybe the the app and the practitioners using the app would benefit from maybe getting a qualitative understanding from their medical history”, C005   - “if all of sudden like you’ve broken your leg, I’m guessing there’d be no way to really tell the app that, so it might start, the app might start flagging things when it’s not actually needed, if that makes sense”, S008   Sleep monitoring   - “It’d be good because it would show me, like say I hadn’t slept enough, it would tell me you need some more sleep”, C006 - “A device would be very very useful because you can’t really explain sometimes why you have negative feelings and it might be really useful to say ‘oh well look, I was feeling negative at this point in the year and I was sleeping, you know like, a lot more than this other point where I slept less’ so I think yeah”, E001 - “See I have something wrong with my insomnia, so that would be more ideal”, E006 - “I think it’d be interesting to know your, going back to the Samsung watch, the sleep things, I think it would be interesting to know how many times I wake up during the night”, G007 - “his is why you’re sleeping too much or…it would just be interesting from that point of view”, E004   Precise location data helpful in an accident, emergency or crisis   - “If I fell somewhere aye I could actually contact the place and they could see it on the sort of satellite navigation system or something”,, E003 - “If you get hurt or anything they can find you easier”, E005 - “It can tell you that you’re having a heart attack so an ambulance can come and get you”, G010 - If “something happened to me”, K006 - “That would be useful information because at least then whoever needs to know where… people are, it’ll be useful then, especially if they’ve got mental health problems or asthma or something like that and something flares up, at least they know where they are”, M007 - “I’d feel safer, it feels like you know if I do need any help if I do need to go into hospital or if I do fall ill, you know obviously then you know where I am and you know how to locate me kind of thing yeah”, M014 - “With people with suicidal thoughts and initiations and ideations, you know pinpoint [location] would be great cos you’d know where they are”, S005   Precise location data helpful if someone goes missing for reasons related to their mental health   - “With my psychosis, it would tell me where to drive and go, so I was going all random locations, it would have been good for my parents to like, hang on now he’s down [location] how the heck did he get down there, laughs all of a sudden, he’s in a psychotic episode. It is good for that reason”, C007 - “I wander a lot when I was ill, I was always taking trains to places where I didn’t know anybody there and stuff… [wife]’s just frightened I’d do that again. So, I think I will put a tracker on my phone… and then she’s reassured that I’m probably in the area”, G007 - “I actually disappeared or not, I end up going to I don’t know, to London or even get in a plane somewhere and not knowing what I was doing then that would be really useful because obviously when you're going though an episode you're not, you're not really in your right mind you're, you're not really kind of functioning properly. So if, if it kind of got flagged up that you basically just disappeared then having some kind of tracking or knowing a distance of how far you've gone I think that would be really useful”, S004 - “I had a dissociative episode and lost like 7 hours and because of the app, I was able to track where I had been, which was really useful for me”, S008 - “Yeah… I mean I guess its helpful in a way say like you got lost and you needed to send someone your location then…But other times its like you’re going about your usual day and it’s tracking you, I don’t really like that”, M012 |

### S6. Summary of views on specific passive sensing data types

During the interview, participants were shown a list of data types that can be collected from a smartphone or wearable via passive sensing (see topic guide Figure 3: supplementary material S2) and asked for their views on these and whether any raised particular concerns. Comments on each data type are summarised below, with illustrative quotations.

The table does not include quotations from participants who agreed to everything on the list of data types, or a sub-set of the list without giving a specific reason why they found these data types acceptable. For example, “Heart rate yep, I agree, light levels, yes I agree. Eh… number and length of phone calls, yep I agree, step count I agree, eh… yep messages” (E001). Similarly, the table does not include quotations from participants who disagreed with all data types because they disagreed with passive sensing as a concept or were uncomfortable about the volume of different types of data gathered. For example, “The sleep, plus the minutes active, plus all the rest of them, just, that’s the Matrix. Oh my head” (K008).

| **Data type** | **Summary and illustrative quotations** |
| --- | --- |
| **Location, n=54** | Covered in detail in the main text, spanning several sub-themes but especially 1.3, 2.1, 3.1, 3.3, and 4.3. |
| **Number of phone calls and text messages**  **(n=14** | Some participants saw the **value of using phone call/text frequency as a proxy for socialisation**: “I’m interested myself to see if I text more or less when I’m down” (E004); “I tend to retreat when I am struggling, I dare say that would probably show up” (G001). However, not everyone understood the purpose of gathering this data. It will be important to explain the specific purpose of gathering call/text frequency to potential DRM users.  Some participants did not want the number of calls/texts to be gathered (“I just don’t like it”, M001), considering it “quite specific to you and a bit more personal… feels a bit like you’re being monitored” (K004) and “a little bit of a privacy breach” (G008). One participant highlighted the **importance of being clear that the system only gathers *number* of calls/texts rather than their *content***:  *“Would have to be explained…that you’re not listening into the phone calls or you’re not reading the text messages”, C007*  Similarly, it was clear from other interviews that some participants initially thought we were asking about participant’s views on how it would feel to have the *content* of calls/texts gathered, rather than just the number of calls/texts, “I would be a bit sceptical because…do you listen to the conversation with friends or is it just the call that is monitored if you call a friend?” (M005). Others sought reassurance that their “phone calls and your text messages were still private” (E004), stating that they “would be less comfortable if it was showing actual message content” (G001). |
| **Sleep, n=10** | Covered in detail in the main text. See sub-theme 4.3. |
| **Heart rate, n=10** | Several participants commented that measuring heart rate would provide a useful indicator of **physical health status**, which could be potentially helpful in an emergency or to prompt them to make lifestyle changes.  *“If someone's heart rate is going up and I'm sure it's already being used like that. You'd be able to find the location and… rush help to them”, K005*  *“It's important to measure my heart rate I think… if it gets dangerously high or something”, S003*  *“Could cover your health with the heartrate”, E003*  *“Heartrate…could make me say well you should go to the gym more or something like that”, E003*  *“Something up with my heart or something I’m sure it would show up, and need to go and see the doctor and go and get my heart checked”, E004*  *“Maybe for me it would be like step count, calories used up, heart rate, just help you keep your eye on your physical health as well, so that would be useful”, M010*  *“It’s the purpose of owning that item is to collect physiological symptoms”, M008*  *“All the health things that you said at the beginning, the heart rate and all that stuff, I would be interested in that”, M001*  *“I think it could look at illnesses quicker that, perhaps, you might get. But then again, you know, it’ll make you a hypochondriac as well, if your heart rate is a bit up that day, you’re thinking to yourself, oh am I going to have a heart attack and...”, K002*  However, as the final quote (K002) warns, collecting heart rate information may fuel health anxiety.  Not many participants commented on **privacy** in relation to heart rate information, although one participant (K009) considered it more personal than other data types, but also said they would be happy for it to be gathered and another (C007) noted that it may be used to infer information about drug use:  *“The only personal one is sleep and maybe obviously your heart rate. Yeah, that’s okay to me”, K009*  *“His heart rate’s gone up a lot since he’s gone to see [fictional name], if you know what I mean, laughs if it was drug use or something”, C007*  One participant commented that passively sensed heart rate information would be **more objective than self-report**:  *“Well, it’s more accurate of the information it can gather because if someone’s asking you, do you think like your heart rate is elevated or lowered more than usual and there is no interpretation, it’s just automatic machine gathered information, whereas if you’re going to input it yourself you might be wrong, you might be exaggerating or something like that”, M008*  Although another warned that **using heart rate as a proxy for stress would have the potential for false negatives** due to individual differences:  *“Cos I might be writing that I’ve had a really horrible day today and you’re thinking ‘oh she’s really stressed’ but my heart rate would show that actually…’cos I think I’ve got quite a low heart rate”, E004* |
| **Bluetooth, n=4** | A few participants expressed concerns about Bluetooth revealing their identity or location or allowing hacking.  *“Gives you the exact location”, K008*  *“Someone could hack you”, K006*  *“Part of my psychosis is to turn Bluetooth off, so if I’m on a bus or anything, no-one knows what my Bluetooth phone is”, G007*  Nevertheless, one of these participants acknowledged that “I know deep down [Bluetooth] is not tracking me stuff” (G007) but they still felt nervous about it.  One participant, who had demonstrated some confusion earlier in the interview about Bluetooth data, said they found it difficult to give an opinion because they did not understand well enough how Bluetooth worked or would be useful in DRM:  *“I’m not 100% sure because I don’t really understand”, M012.*  This underlines the importance of explaining clearly, during informed consent for using a DRM system, how Bluetooth data would be used to ensure that DRM users understood what they were agreeing to. |
| **Skin temperature, n=4** | Only four participants commented directly on the idea of gathering skin temperature data. One expressed discomfort with the idea (G008), and two made ambivalent comments: “Well, skin temperature, I think that’s a bit vague, isn’t it?” (K002) and “I’m not sure how important the skin temperature” (K004). One participant suggested that skin temperature would be useful for spotting alcohol poisoning: “Your skin temperature if you’ve got a…involved in alcohol you know, alcohol poisoning” (E003). |
| **Step count, n=3** | Three participants commented specifically that step count was acceptable or of interest because people often like to know how much activity they have done, especially as this can impact their wellbeing: “I know if I’m out and about, then that helps my wellbeing, that helps me feel good, but sometimes that doesn’t happen. So it might just alert me to think, okay, how can I … increase my activity levels, or just think about what have I been doing that has reduced it or increased it” (K004). |
| **Calories, n=3** | Only three participants commented specifically on calories, with one expressing discomfort with the idea (G008), one expressing puzzlement about how this might be calculated, “Calories used up; I think that’s a bit abstract… how can you really analyse how many calories have been used” (K002) and one was “not sure how important” measuring calories used up would be (K004). |
| **Minutes active, n=2** | Some participants commented that they did not know what this meant:  *“Minutes active, I dunnae ken what that means”, E003.*  However, one participant who understood that it meant time spent active rather than sedentary, thought that activity level may be valid estimate of symptoms:  *“In the past few months I’ve been active on my indoor bicycle, my exercise bicycle, and I do about 5kms, and at the minute its going very well…but when I get low I don’t feel like doing it and it tails off, it can disappear for a time which is not good because the exercise had definitely helped with my mood swings”,, E008* |
| **Audio, n=2** | Only two participants commented on audio. One spontaneously suggested that gathering the level of background noise may be valuable:  *“If there were something saying like what the background…’cos sometimes you go places and there’s like a general background noise that’s just so loud. When you’re in traffic and stuff, that might…that might be useful?”, E001*  However, another participant warned of the potential for false positives due to their home being generally very noisy:  *“My house is really noisy… I've got three boys, and it gets so…sometimes I might shout at them, shouting, oh stop that. Because sometimes, when you shout, then they do the right thing, or refrain from the wrong thing they are doing”, M006* |
| **Air temperature, n=2** | One participant, M012 expressed surprise that their phone tracked air temperature, whereas a second participant thought that “air temperature absolutely would be useful” (E001). |
| **Light levels, n=2** | “Makes me feel a bit uneasy because its like how do they know that I’m inside or outside”, M012 |

### S7. Answers to the question “Would you want to see a copy of the passive sensing data that the system collects?”

#### Reasons for not wanting to see a copy of the passive sensing data

A minority of participants (5/27 participants who commented) said they would not want to regularly see a copy of passive sensing data that the system collects. Reasons given were:

- “I maybe only wear my Fitbit if I’m doing exercise, cos I don’t want to have some sort of micromanaging thing…I don’t think I would want to be notified about all the passive information that is being gathered cos most people… don’t want any external information to influence…how they are doing their day-to-day tasks”, G008. See also Theme 2.3.
- “I think if I really wanted to I’d request it more than I’d want to be shown it”, G008
- Not necessary: “I go for a check-up, like once a quarter, I'm good”, K005
- Would want to see mood tracker info but not passive info: “No, I don’t think so. But maybe a mood tracker kind of thing, so that I can look myself to see where change is. I think that is pretty important”, M001

#### Reasons for wanting to see a copy of the passive info

Most participants (22/27 participants who commented) said they would want to see a copy of the passive sensing data that the system collects. Reasons given were:

- To have my own record, spot patterns, or because it’s interesting
  - “I like having records”, E003
  - “If you keep it… for…for future reference and say well that happened there and that happened there, so if a change from that to that…it might help…to have another look kinda like at a health problem”, G006
  - “Yes, I would definitely like to see the patterns of my health and behaviour”, M005
  - Not because worried about passive sensing, just interested: “Probably not ‘cause I was worried, I’d just be interested, just to check really” K001
  - “I’d be interested in what’s going on”, M003
  - “Because it might be helpful and useful. Yeah, yeah. So it would be helpful to see the information. Yeah”, M007
  - “Yeah ‘cus then you can like you know see how much progress you’ve made…”, M012 – talking about progress in meeting physical health goals
- Want to see the same info the team are getting
  - Participant E003 wouldn’t be worried about passive sensing “as long as em…I was able to see it myself” so that they knew where the doctors were getting the information.
  - “I wouldn’t like them to see all this information and then know stuff about me that I don’t even know”, K007
  - So that can see what others see. The participant compared this with scenario with another occasion when had sent a form off for an appeal related to work: “I ticked the box to see the report because it goes back to my work and I know exactly what is going on that way”, G007
- Spontaneously suggested in response to what would help people feel more comfortable with passive:
  - “Just give people, if possible, the potential to share the information with the person”, K001.
  - “I think if there was a way to view what information has been sent, even if it’s just raw data then I think being able to have the statistics on what’s been sent, I think that helps people…it helps people know that what they’ve agreed is what’s being sent and there is nothing leaking that they’re not aware of. So I think even if you just had information like a total step count or minutes or hours of location transferred or stuff like that, I think that helps people…it’s like a level of verification on what they’ve agreed to”, M008
- Would be ok to see infrequently but not all the time: “Maybe not like all the time, maybe like a review after like a quarterly review, just to say like these are your percentages where you’ve been out, I wouldn’t, it’s not something I’d look back on, or want to look back on each week and go like what did I do last week or the week before”, M011

#### How would you want to receive the passive sensing information?

- Don’t mind – C001
- In app – C003, G005, G007, K003, M005
  - “Sort of in the app or a text message around the general information that’s been collected”, C003
  - “Ah that could be…I think if I could choose, you know like if…in the app, would you like to see in the app or in email or print it, I like to…but I… most important thing it would be in the app”, G005
  - “Keep it all in once place”, G007
  - “I think via app because it’s instant…And it can be reviewed or stored for archiving, and assessments…”, K003
  - “I think seeing it…if everything is integrated in the app, it would be much more accessible, you know, easily accessible. I think that’s easier that way when you can see everything in the app”, M005
- Email – K001, K007, M004, M007
- Post – G006, K009
  - “I don’t have email at the moment”, K009
- In person with a researcher, M003:
  - “Best way that I think…is, if the researcher comes round and you can sit down, you can both look at it on a laptop or on a smart phone and then talk through it, like I did with the [name] project. I don’t think it’s something you can really comprehend in your home or by yourself. You need somebody just showing you what the highlights are”, M003

#### How often would you want to receive the passive sensing information?

- Ongoing basis, checking when you want to - M004, M012, G003
  - “Just as and when”, M004
- At least twice a day – G005
  - “At least when I wake up and when I go to bed” – G005
- Every couple of days - M007
- Weekly – C001
- Fortnightly – G007
- Every 3 months – G004, M011
- Once a month – K007
- Just once – G006

### S8. Participants’ preferences regarding frequency of passive sensing consent renewal


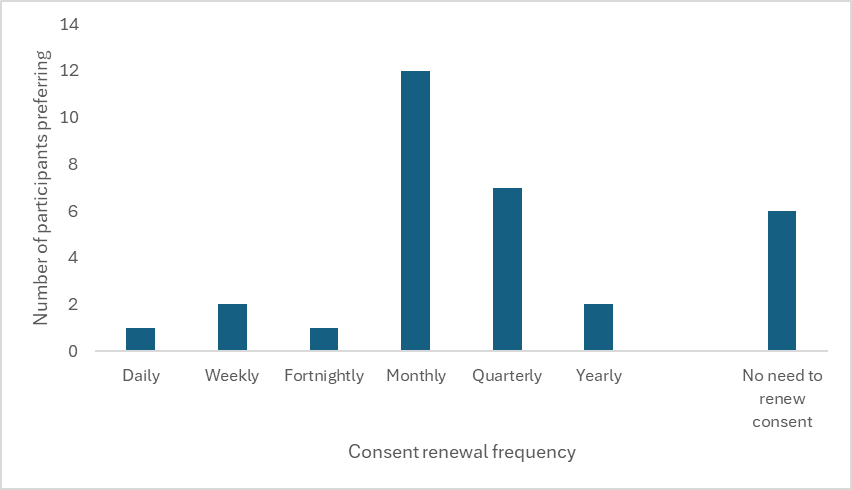

Supplement: Connect_SU_quali_Paper_1_-_supplementary_material_02_05_25_sbaf126 [file connect_su_quali_paper_1_-_supplementary_material_02_05_25_sbaf126.docx]
